# An ensemble deep learning framework for emotion recognition through wearable devices multi-modal physiological signals

**DOI:** 10.1038/s41598-025-99858-0

**Published:** 2025-05-18

**Authors:** Durgesh Nandini, Jyoti Yadav, Vijander Singh, Vijay Mohan, Saurabh Agarwal

**Affiliations:** 1 Department of ICE, Netaji Subhas University of Technology, Sector 3, Dwarka, New Delhi, India; 2 Department of Mechatronics, Manipal Institute of Technology, Manipal Academy of Higher Education, Manipal, Karnataka 576104 India; 3 School of Computer Science and Engineering, Yeungnam University, Gyeongsan, 38541 Republic of Korea

**Keywords:** Emotion recognition, Wearable devices, Deep learning, Ensemble architecture, EMOGNITION database, Physiological signals, Machine learning, Biomedical engineering

## Abstract

The widespread availability of miniaturized wearable fitness trackers has enabled the monitoring of various essential health parameters. Utilizing wearable technology for precise emotion recognition during human and computer interactions can facilitate authentic, emotionally aware contextual communication. In this paper, an emotion recognition system is proposed for the first time to conduct an experimental analysis of both discrete and dimensional models. An ensemble deep learning architecture is considered that consists of Long Short-Term Memory and Gated Recurrent Unit models to capture dynamic temporal dependencies within emotional data sequences effectively. The publicly available wearable devices EMOGNITION database is utilized to facilitate result reproducibility and comparison. The database includes physiological signals recorded using the Samsung Galaxy Watch, Empatica E4 wristband, and MUSE 2 Electroencephalogram (EEG) headband devices for a comprehensive understanding of emotions. A detailed comparison of all three dedicated wearable devices has been carried out to identify nine discrete emotions, exploring three different bio-signal combinations. The Samsung Galaxy and MUSE 2 devices achieve an average classification accuracy of 99.14% and 99.41%, respectively. The performance of the Samsung Galaxy device is examined for the 2D Valence-Arousal effective dimensional model. Results reveal average classification accuracy of 97.81% and 72.94% for Valence and Arousal dimensions, respectively. The acquired results demonstrate promising outcomes in emotion recognition when compared with the state-of-the-art methods.

## Introduction

Emotions represent momentary and coherent reactions, encompassing verbal, physiological, behavioral, and neural responses to internal or external stimuli. Thus, emotions constitute a fundamental dimension of human existence, exerting significant influence on one’s thoughts, behaviors, decisions, and thus overall well-being or quality of life. Unhealed reactions may give rise to various manifestations, such as depression, anxiety, stress, fatigue, sleep disorders, drowsiness, loss of appetite, and alterations in activity and sleep patterns. Emotionally contextual services can greatly improve user experience through precise, contextual recommendations. Affective computing identifies human emotions through interaction cues and generates emotionally intelligent responses for machines with emotional capabilities.

The literature uncovers the utilization of two emotion recognition models, namely discrete and dimensional models. The former identifies fundamental discrete emotional states acting as individual entities that can be recognized and assigned specific labels. Such models include basic emotions like happy, sad, etc., thereby being easy to comprehend and suitable for preliminary emotion identification. Conversely, the latter accommodates a wide spectrum of emotional intensities via continuous scales for valence (V) and arousal (A) dimensions. The dimensional models are ideal for detailed emotional portrayal, where the V and A dimensions depict the degree of pleasantness and excitement. However, it should be noted that the categorical model offers high comprehensibility compared to the dimensional model due to ease of use. Some notable discrete models include Paul Ekman’s model, Plutchik’s Model, and the Vector Model, while the dimensional models are Russell’s circumplex model^1^. The emotions can be analyzed by examining variations in diverse non-physiological or physiological modalities. However, relying on non-physiological methods in emotion detection can lead to failure in accurately identifying a person’s genuine emotional state, as individuals can conceal or feign their emotions. On the contrary, the physiological modalities demonstrate a superiority over non-physiological methods in emotion recognition. Emotional variations are mirrored in physiological changes, emphasizing usability and importance in emotion recognition^2,3^. Some of the widely used physiological modalities are electroencephalogram (EEG), electrocardiogram (ECG), Galvanic Skin Response (GSR), Heart Rate (HR), Blood Volume Pressure (BVP), and respiration rate (RSP). Numerous researchers have employed EEG signals for emotion detection^4,5^. However, EEG’s applicability is restricted to a stationary position, lacking the advantages of wearable devices. On the other hand, the advancement in miniaturization in wearable device technology has facilitated the repeated or continuous digital measurement of parameters, often in real-time, providing distinct advantages over EEG in terms of mobility and versatility^6^. Wearable technology facilitates biofeedback and physiology tracking, enabling constant accessibility to crucial information^7,8^. The widespread availability of several advanced wearables, including Empatica wristband, Apple iWatch, Fitbit, Samsung Gear, etc., facilitate non-intrusive health monitoring, including epilepsy detection^9^, emotional care for autism spectrum disorder (ASD) impacted children^10^, Rett syndrome (RTT) detection^11^ and stress detection beyond laboratory environments^12–17^. Thus, it underscored the potential of wearable sensing devices in the recognition of human emotions.

Section Related work discusses related work in detail, including the contribution of the present work. Section Methodology is the methodology, which includes a detailed discussion of the framework for the proposed emotion recognition system. Section Results and discussion contains experimental analysis and results. The work is summarized with limitations and future direction in Section Conclusion.

## Related work

Recent advancements in artificial intelligence and pervasive computing have significantly propelled research in emotionally intelligent human-computer interaction (HCI) systems. The availability of economical wearable devices capable of capturing various physiological signals caters to rich HCI experiences. Various researchers employ the combination of multiple physiological signals in the design and development of emotion detection systems. Wang et al.^18^ explores geometric algebra-enhanced networks and Zhu et al.^19^ brain-inspired hierarchical models for emotion recognition. Zhu et al. also introduced a non-contact multimodal system assessing emotions via voice, text, and facial expressions^20^. Other approaches leveraging tensor decomposition and self-supervised learning improved multimodal emotion recognition^21^, complemented by comprehensive reviews of key technologies^22^.

Moreover, Domínguez et al.^23^ developed an emotion recognition system using photoplethysmogram (PPG), HR, and GSR signals, achieving excellent accuracy for three discrete emotions primarily through GSR features analyzed with random forest (RF) and support vector machine (SVM). Alqahtani et al.^24^ presented an Intelligent Tutoring System utilizing EEG, ECG, and EMG signals, achieving a 74.21% F1 score using SVM and EEG-based Mel-frequency cepstral coefficients (MFCC) features to substitute human tutors with adaptive physiological signal analysis. Mert^25^ classified EEG, EOG, EMG, GSR, RSP, PPG, and temperature signals from the DEAP database using Short-Time Fourier Transform (STFT), Variational Autoencoder (VAE), and Naïve Bayes (NB), achieving around 79% accuracy for the binary VA model. Ismail et al.^26^ compared ECG and PPG for emotion detection, finding ECG better for arousal and PPG superior for valence, reporting a maximum accuracy of 64.94% while analyzing six fundamental emotional states. Nandi and Fatos^7^ employed federated learning for multi-modal physiological signal classification in the DEAP database, ensuring participant privacy. Nita et al.^27^ utilized a seven-layer convolutional neural network (CNN) to augment and classify ECG signals from the DREAMER database, achieving accuracy rates of 95.15%, 85.56%, and 77.54% for the 3D VAD model, respectively. Baghizadeh et al.^28^ analyzed ECG signals from the MAHNOB-HCI database using Poincare maps and SVM, achieving accuracies of 82.17% and 78.07% for valence and arousal, respectively.

Conversely, Bulagang et al.^29^ used ECG and GSR signals from the AMIGOS dataset with CNN-based feature extraction to classify valence and arousal, demonstrating improved precision. Wei et al.^4^ combined ECG and EEG signals, achieving 84.6% accuracy with SVM for five discrete emotions. Baig et al.^2^ achieved 90% accuracy using SVM on ECG signals, focusing solely on arousal. Ferdinando et al.^30^ reported accuracies of 64.1% for valence and 66.1% for arousal with ECG signals using the KNN algorithm, whereas A. Goshvarpour et al.^31^ achieved 80.24% accuracy with SVM on ECG signals for emotions including happiness, sadness, relaxation, and fear. Muñoz-Saavedra et al.^32^ developed a wearable device using PPG and GSR signals with a multi-layer perceptron (MLP) neural network, achieving accuracies of 84.27% for valence and 91.82% for arousal, although reproducibility remains a concern. Kacimi and Adda^33^ used DEAP physiological signals with principal component analysis (PCA) and RF classifiers, obtaining approximately 75.5% accuracy for valence and arousal. Wan et al.^34^ proposed a real-time virtual-reality (VR)-induced emotion model using EEG, PPG, GSR, and skin temperature (SKT) signals, achieving 89.68% accuracy for valence and 90.11% for arousal using the LightGBM classifier; however, the model emphasized wearability at the expense of capturing emotional complexity.

Further, Kumar et al.^35^ utilized AMIGOS and ASCERTAIN datasets with EEG, ECG, and GSR signals, employing RF, k-nearest neighbor (KNN), and logistic regression(LR) classifiers. RF achieved up to 93.15% accuracy for valence-arousal classification using EEG-GSR, while LR reached 100% accuracy on ECG signals in ASCERTAIN; however, EEG usage reduced wearable practicality. Wang et al.^36^ applied Bi-LSTM with attention mechanisms on EEG signals from SEED and DEAP datasets, achieving accuracies of 98.28% and 92.46%, respectively, but noted limited wearability, suggesting integration of peripheral signals for enhanced performance. Chen et al.^37^ developed a wearable fNIRS-EEG Temporal Convolutional Networks (TC-ResNet) system, achieving 99.81% accuracy, surpassing unimodal methods, yet testing a limited range of emotions, which affects practical wearability. Sedehi et al.^38^ combined EEG and ECG data from the MAHNOB-HCI dataset using a residual network (ResNet-18) CNN, achieving 91% accuracy, but focused only on happiness and sadness. Lee and Yoo^39^ utilized Stacked Autoencoder (SAE) and long-short term memory (LSTM) methods on ECG, GSR, and skin temperature signals, achieving 99.4% accuracy but exclusively studied negative emotions. Lee et al.^40^ employed 1D-CNN on PPG signals from the DEAP dataset, obtaining approximately 75% accuracy in short-term emotion recognition. Sepúlveda et al.^41^ achieved accuracies of 88.8% for valence and 90.2% for arousal using wavelet transform and ensemble classifiers on ECG signals from the AMIGOS dataset. Irshad et al.^42^ implemented transfer learning on signals collected using Empatica, Emotiv EEG, and Biosignalplux RespiBAN devices, achieving around 73% accuracy. Cosoli et al.^43^ and Zhao et al.^44^ used Empatica E4 wristband signals, obtaining approximately 75% accuracy, but highlighted limitations due to small participant samples.

Literature exhibits limitations in wearability, scalability, and emotion diversity, primarily due to reliance on multi-channel sensing devices, constrained physiological signal integration, and a limited range of affective states, underscoring the need for more adaptable, multimodal, and real-world deployable emotion recognition systems. The suitability and validity of wearable sensors in emotion recognition lack comprehensive exploration. Using common wearables is vital for result reproducibility, providing a standardized data collection platform, ensuring uniformity, facilitating result comparisons, and promoting broad applicability. A literature gap exists in evaluating specific wearable devices, emphasizing the need to explore emotion detection through standard wearable sensing devices.

Recently, ensemble DL architectures have demonstrated promising results in several applications, including earthquake casualty prediction^45^, fake news instance detection^46^, etc. Ensemble or specifically stacked ensemble models harness the strengths of individual models to bolster detection capabilities. For example, Iyer et al.^47^ studied the implementation of CNN, LSTM, and a combination of CNN-LSTM to identify emotion through DEAP and SEED datasets with an average classification accuracy of about 4%, which is superior to individual models on the SEED dataset. Similarly, Zheng et al. proposed a hybrid GRU-LSTM model^48^ for parking occupancy prediction, motivating the design of the proposed hybrid or ensemble DL model. Pudumalar and S^49^ achieved 95% accuracy in plant disease detection, surpassing individual models with their ensemble CNN and VGG 16 model. Islam et al.^50^ demonstrated the combined efficacy of GRU and LSTM models for FOREX market forecasting. Similarly, Cui et al.^45^ emphasized stacking-based ensemble learning advantages in earthquake casualty prediction. Mohammed et al.^51^ asserted that stacking ensemble techniques provide superior predictive power, improved data understanding, and increased precision. Thus, the present work is motivated to investigate the stacking-based ensemble learners for emotion recognition.

The literature survey reveals that most emotion detection systems rely on EEG signals, which are often impractical and vulnerable to noise and artifacts. It motivates the utilization of peripheral physiological signals over EEG signals. It can provide a contextual, rich, and comprehensive approach to designing an emotion detection system. People are increasingly choosing user-friendly fitness trackers to continuously monitor health parameters, such as stress, highlighting the need for a comprehensive examination of these devices in emotion recognition. However, signals acquired using these devices suffer from motion artifacts, and the dynamic nature of emotions makes it challenging to design a reliable emotion recognition system using wearable devices. The literature survey indicates that the sequential character of LSTM enables it to capture temporal patterns, hence facilitating the recognition of reliable emotions even in the presence of motion artifacts. The hierarchical feature extraction method effectively addresses the difficulties caused by motion artifacts in physiological signal data. These models do not rely on explicit feature extraction and can also easily handle high-dimensional data. Models are also well suited to analyze sequential and time series biomedical data.

The existing studies tend to lack specific and targeted analysis dedicated to a particular wearable sensing device. Research has yet to investigate three sets of signal combinations from Samsung Galaxy Watch, Empatica E4, and EEG headband MUSE 2 wearable devices for emotion detection. Hence, the present study employs the publicly available EMOGNITION database consisting of various physiological signals elicited during emotional video stimuli. The database consists of an accelerometer (ACC) (3x), BVP (2x), EEG, Electrodermal Activity (EDA), gyroscope (GYRO) (2x), HR, and SKT captured using three wearable devices: Samsung Galaxy Watch, Empatica E4, and EEG headband MUSE 2 device. The signals obtained from each device are pre-processed and examined for a uniform class balance by employing the Synthetic Minority Oversampling Technique-Tomek (SMOTE-Tomek) technique. An ensemble stacked LSTM-GRU DL architecture is utilized for both binary and multi-class signal classification. A thorough analysis of the performance of emotion detection models encompassing both discrete and dimensional models is conducted. The results are then compared with the existing works of literature, demonstrating the promising potential for integration into various emotion recognition-based applications. The key contributions of this work are listed below:

- This is the first work to suggest designing discrete and dimensional emotion detection models employing wearable technology and physiological signals to understand emotions comprehensively.
- The work utilizes the multimodal ACC (3x), BVP (2x), EDA, EEG, GYRO (2x), HR, and SKT physiological signals gathered from the Samsung Galaxy Watch, Empatica E4, and EEG headband MUSE 2 wearable devices from the EMOGNITION database to design a robust emotion recognition system.
- The SMOTE-Tomek technique is applied to ensure an optimal model fit by balancing the class distribution. SMOTE generates synthetic samples for the minority class to reduce imbalance, and Tomek removes ambiguous samples near class boundaries to improve class separation.
- The system design incorporates the LSTM-GRU ensemble stacked DL architecture to capture temporal patterns within emotional data sequences using the combined strengths of individual models.
- The experimental results show the efficiency of the Samsung Galaxy smartwatch in determining emotions. The proposed scheme achieves better performance compared to the existing method, highlighting the capability for real-time emotion detection using physiological signals acquired from wearable sensors.

## Methodology

**Fig. 1:**
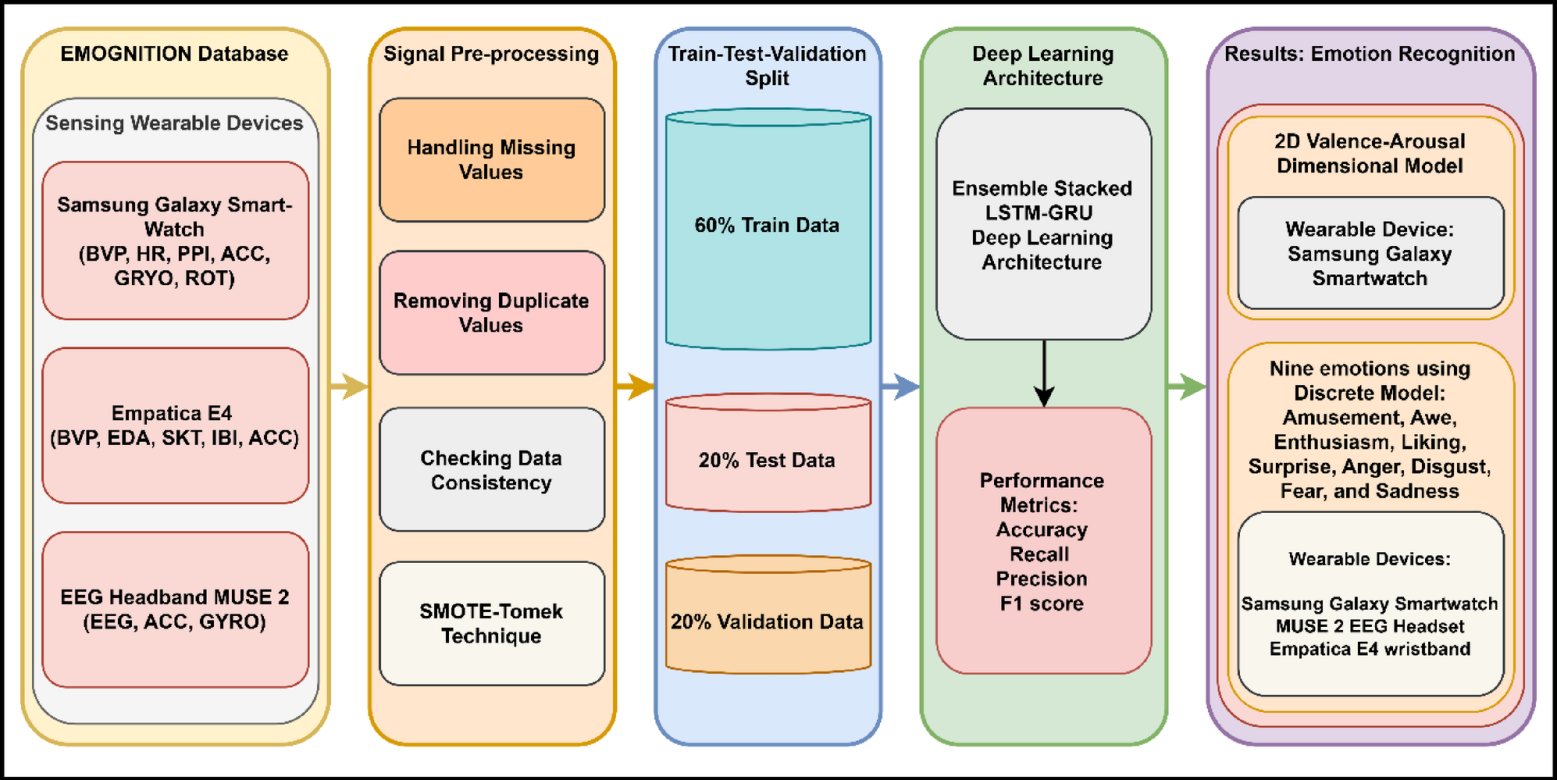
The framework for the proposed emotion recognition system.

Over the last few years, small wearables with capabilities for health and fitness have taken off and changed the way we track vital human health parameters. This opened a new era of human and computer interaction with the ability to have emotionally intelligent, contextually aware conversations. In this area, accurate emotion detection is said to be key to nuanced human-technology interactions. In this paper, a formalized framework for emotion recognition is presented through wearable technology, described in five sequential blocks, which, as a whole, allow for an insight into emotional dynamics via discrete and dimensional-based models. Figure 1 outlines the methodology used in this article, starting from Block 1, involving recording physiological data by three different wearable devices: the Samsung Galaxy Smartwatch, the Empatica E4 wristband, and the MUSE 2 EEG headband. The EMOGNITION database relies on these devices to deliver a range of physiological signals, including but not limited to BVP, HR, and EDA, all essential for accurately determining emotional states. Block 2 is responsible for the correct pre-processing of the data to make it robust and reliable, also checking for missing values, eliminating duplicate rows, consistency checks, etc. The SMOTE-Tomek method is applied to balance out the different emotion classes, thus preventing class imbalance and ultimately improving the final model result. In Block 3, data breakdown is displayed: 60% of the data is utilized for training, and then 40% is divided equally between testing and validation. This data split yields a sufficiently rich evaluation setting that tests generalization on unseen data well. Block 4 offers the beating heart of the quant engine: a DL ensemble of LSTM and GRU models. By combining the advantages of both models, this hybrid structure can effectively capture the temporal dynamics of the system and identify potential emotional patterns embedded in the data sequences. Lastly, block 5 gives a detailed analysis of the proposed framework. With the discrete model, nine basic emotions were identified across the collected data: amusement, awe, enthusiasm, liking, surprise, anger, disgust, fear, and sadness. The confirmation of distance between data points, using the 2D Valence-Arousal (VA) dimensional model with the Samsung Galaxy Smartwatch, carries more nuanced insights regarding the intensity of emotional experience.

This research, with a structured approach, proves not only the predisposition of wearables for emotion recognition but also provides an invaluable addition to the rapidly changing landscape of affective computing, reinforcing activity in an area that could greatly benefit HCI. Given that emotions are naturally separate and dimensional, this study presents a conceptual framework that makes the application and understanding of emotional-aware technology in the real-world context a lot clearer. Each block detail is defined below.

### Database

The proposed emotion recognition model exploits the publicly available EMOGNITION database^52,53^, collected from July 16 to August 4, 2020, in the UX Wro laboratory at Wroclaw University of Science and Technology. The database consists of 43 subjects’ physiological signal responses collected through three wearable devices while watching short film clips elicitation. The 43 participants comprised 21 females and 22 males aged between 19 and 29. The participants self-reported the emotions experienced during the short film clip elicitation using both the discrete and dimensional emotional model representations. The short films are curated and targeted to arouse nine discrete emotions: amusement, awe, enthusiasm, liking, surprise, anger, disgust, fear, and sadness, and three affective dimensions: valence, arousal, and motivation. The database experimental procedure included ten iterations of showing the participants a 2-minute washout clip to obtain the physiological baseline, then a 1–2-minute emotional stimulus film clip, and the two self-assessments. The participants categorically self-reported nine discrete emotions, indicating intensity on a single-item scale from “1: not at all” to “5: extremely”. The participants also rated valence, arousal, and motivation dimensions of the dimensional model via the Self-Assessment Manikin (SAM) single-item rating scale, spanning from “1: least intensity” to “9: most intensity”. The ACC (3x), BVP (2x), EDA, EEG, GYRO (2x), HR, and SKT physiological signals are collected using the EEG headband MUSE 2, a wristband Empatica E4, and a smartwatch Samsung Galaxy SM-R810 wearable devices. The MUSE 2 device contains subjects’ accelerometer (ACC), EEG, and gyroscope (GYRO) signals. The EEG channels used for data collection are AF7, AF8, AF9, and AF10. Simultaneously, Empatica E4 records interbeat interval (IBI) of the BVP and ACC signals, blood volume pulse (BVP), electrodermal activity (EDA), and skin temperature (SKT). Similarly, the Samsung Galaxy watch measures the subject’s ACC, BVP, GYRO, heart rate (HR), peak-to-peak interval (PPI), and rotation signals.

### Signal Pre-processing

The EMOGNITION database utilized in this study includes pre-processed multimodal physiological signals acquired from three wearable devices, namely the Empatica E4 wristband, the Samsung Galaxy watch, and the Muse 2 headset. Among the three wearable devices, the Empatica E4 provides the most heterogeneous number of data samples across different modalities. Thus, in order to facilitate temporal alignment and ensure compatibility for early fusion or data-level fusion within the proposed ensemble LSTM-GRU DL framework, all signal modalities are resampled to a unified time base. It is important to note that data-level fusion is performed for each wearable separately. The early-fusion technique integrates and captures the complementary information present across the multiple modalities at the feature level before the classification process. Since the linear interpolation technique has yielded satisfactory results for HRV feature extraction by preserving the linear trend of the data while introducing minimal artifacts^54^. The work adopts the linear interpolation resampling technique due to its computational efficiency and ease of implementation.

Furthermore, the study thoroughly investigates the signals database recorded using each wearable device to identify any missing, inconsistent, or duplicate data samples. The data consistency in the number of samples across all channels is ensured by standardizing the sample count. The work is designed using both the discrete and dimensional models of emotion detection. The developed discrete emotion recognition system utilizes a binary approach for classification, whereas the dimensional model undertakes multi-class classification. The dimensional model considers three-class classification, i.e., low, medium, and high. Any imbalance in the class distribution for either the binary or multi-class approach is handled using the SMOTE-Tomek Technique^1^.

### SMOTE-Tomek technique

An imbalanced class distribution introduces a skew in the decision boundary, resulting in classifiers that disproportionately favor the majority class and exhibit limited generalization capability for minority class instances. Due to their low frequency, minority samples are often misclassified as noise or outliers, thereby degrading overall model performance. This issue can be addressed either at the data level or through algorithmic modifications. At the data level, oversampling techniques such as the Synthetic Minority Oversampling Technique (SMOTE) are commonly employed to increase the representation of the minority class artificially. SMOTE generates new synthetic instances using the equation:

1 $$\:\text{S}\_\text{n}\text{e}\text{w}\:=\:\text{r}\:\times\:\:(\text{S}\_\text{k}\text{n}\text{n}\:-\:\text{S}\_\text{b}\text{a}\text{s}\text{e})\:+\:{\text{S}}_{\text{b}\text{a}\text{s}\text{e}}$$

where S_new denotes the synthetic sample, S_base represents a randomly selected minority class instance, S_knn is one of its k-nearest neighbors, and r is a randomly chosen scalar between 0 and 1.

The SMOTE approach creates new samples along the line segments joining minority class instances and their neighbors, thereby introducing diversity and reducing the likelihood of overfitting.

2 $$\:{\text{T}\text{o}\text{m}\text{e}\text{k}\_\text{l}\text{i}\text{n}\text{k}}_{\left(\text{a},\text{b}\right)}=\left\{\begin{array}{l}1,\:if\:{\text{C}}_{\text{a}}={\text{C}}_{\text{b}\:}and\:\nexists\:\:C:d\left(\text{a},\text{c}\right)<d\left(\text{a},\text{b}\right)or\:d\left(\text{b},\text{c}\right)<d(a,b)\\\:0,\:otherwise\end{array}\right.$$

where $\:{\text{C}}_{\text{a}}$: sample a belongs to class a, $\:{\text{C}}_{\text{b}}$: sample b belongs to class b, $\:\text{d}\left(\text{a},\text{c}\right)$: distance between sample a and c, $\:\text{d}\left(\text{a},\text{b}\right)$: distance between sample a and b, $\:\text{d}\left(\text{b},\text{c}\right)$: distance between sample b and c,

Conversely, under-sampling methods aim to reduce the number of majority class instances to achieve a more balanced distribution. The Tomek Link method identifies pairs of samples from different classes that are each other’s nearest neighbors. Equation (2) illustrates a Tomek link that exists between two samples, a and b, if a belongs to one class, b belongs to another, and there is no third instance that is closer to either a or b than they are to each other. Such links typically occur near class boundaries and represent ambiguous or overlapping regions. Removing Tomek links helps to improve class separability by eliminating borderline examples. However, while under-sampling may lead to the loss of valuable information, oversampling can increase the risk of overfitting due to redundant synthetic patterns. Thus, to address the limitations inherent in each technique, the study adopts a hybrid SMOTE-Tomek approach, which combines the advantages of both methods by simultaneously enhancing minority class representation and removing noisy or overlapping majority class instances, thereby improving the model’s robustness and classification performance.

In this study, the SMOTE-Tomek method is applied exclusively to the training set to achieve a balanced class distribution. This strategy is adopted to preserve the natural class imbalance in the test set, thereby avoiding data leakage and ensuring unbiased performance evaluation^54,55^. The proposed ensemble LSTM-GRU deep learning model is thus trained on a balanced set of emotion classes, while evaluation is conducted on data that reflects real-world class distributions. This approach enables the model to effectively learn from a uniform class representation during training without compromising the validity of the testing process.

### Classification

Unlike traditional machine learning, DL techniques do not require explicit signal processing, feature extraction, or feature selection techniques to learn the underlying characteristic features for classification. Thus, it provides an advantage over traditional machine learning techniques by eliminating several intermediate steps required to develop a discriminative or predictive model. The DL architecture utilizes several layers of sequential neural networks to learn the characteristic patterns of the input and provide classification results. The traditional recurrent neural network (RNN) architecture finds applications in sequential data analysis, but it faces vanishing gradient limitations. LSTM and GRU are subtypes of RNN architecture that can overcome such limitations and effectively sustain data across lengthy sequences. In this work, the simplified and effective architectures of both LSTM and GRU models are fused using the ensemble stacked technique. Hence, the work leverages the combined strengths of each model for emotion recognition.

**Fig. 2:**
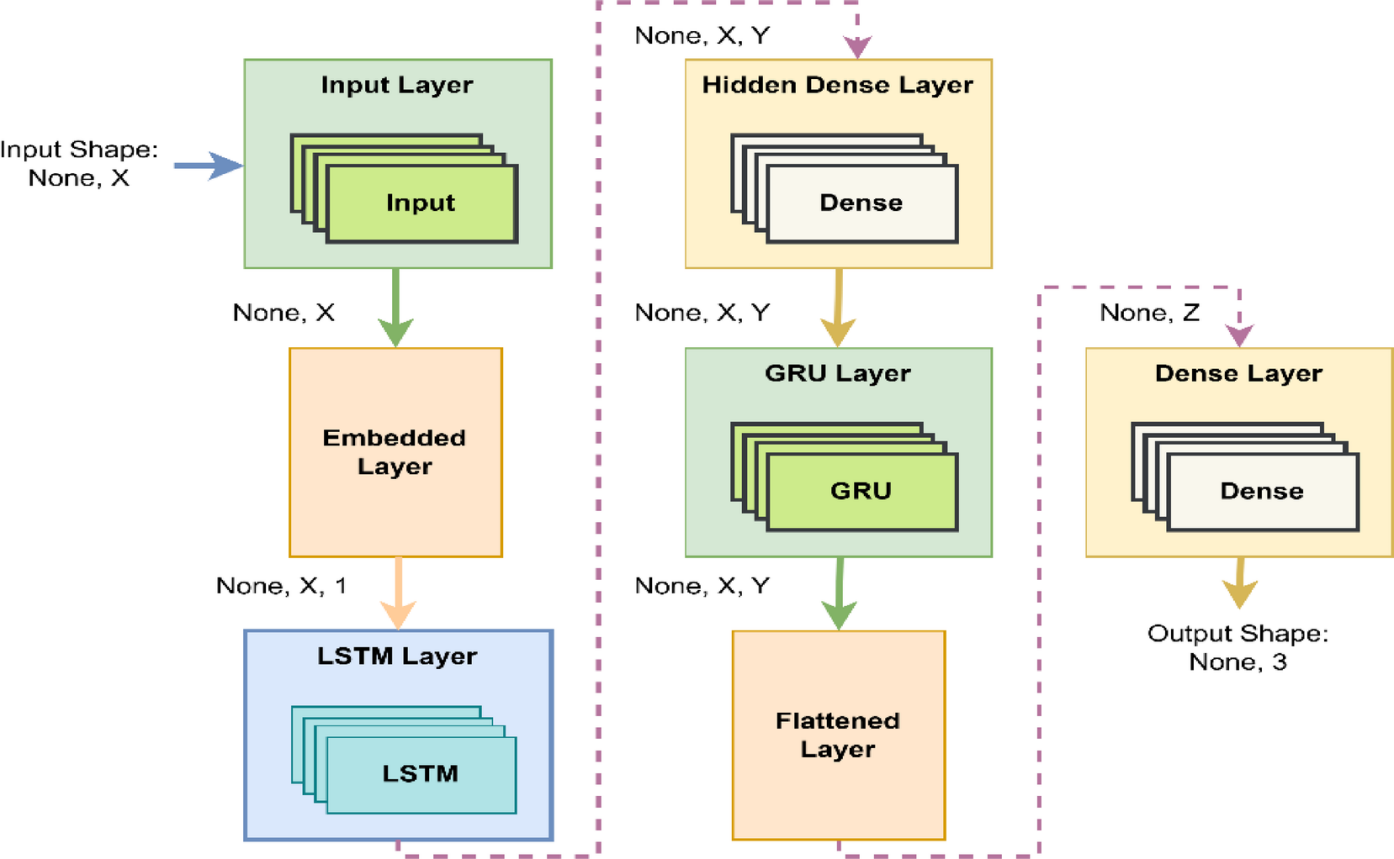
Depicts the ensemble stacked deep learning architecture, where x and y depend on the output shape after each layer.

Figure 2 depicts the envisioned ensemble-stacked DL architecture formed by amalgamating the individual LSTM and GRU models. The developed architecture is tailored to craft an automated emotion recognition system by combining the individual strengths of each model. The work utilises the stacking mechanism, based on the sequential stacking approach proposed by Cohen and Carvalho^56^, where the output of one model is used as input to another to improve sequential prediction. In the proposed architecture, LSTM and GRU layers are arranged in a serial configuration, with the output sequence from the LSTM passed directly into the GRU layer. This design allows the model to take advantage of the distinct characteristics of both recurrent units. The LSTM is effective in retaining long-range temporal dependencies due to its gated memory structure, while the GRU offers a more compact formulation that captures short-term dynamic patterns efficiently. As shown in Cohen’s work, sequential stacking enables the later model in the sequence to utilize the structured context generated by the earlier one, which is especially beneficial for modeling time-dependent data. Hence, the stacking strategy enhances temporal feature learning and allows the model to build richer and more discriminative representations over time.

**Table 1:** Illustrates the parameter structure for the proposed discrete emotion recognition system.

Layer (type)	Muse 2 Headband		Samsung Galaxy Watch		EmpaticaE4 wristband	
	Output Shape	Param #	Output Shape	Param #	Output Shape	Param #
input_1 (InputLayer)	[(None, 26)]	-	[(None, 13)]	-	[(None, 7)]	-
tf.expand_dims (TFOpLambda)	(None, 26, 1)	-	(None, 13, 1)	-	(None, 7, 1)	-
lstm (LSTM)	(None, 26, 32)	4352	(None, 13, 32)	4352	(None, 7, 32)	4352
dense (Dense)	(None, 26, 32)	1056	(None, 13, 32)	1056	(None, 7, 32)	1056
gru (GRU)	(None, 26, 32)	6336	(None, 13, 32)	6336	(None, 7, 32)	6336
flatten (Flatten)	(None, 832)	-	(None, 416)	-	(None, 224)	-
dense_1 (Dense)	(None, 2)	1666	(None, 2)	1090	(None, 2)	450

The physiological signal sequences captured from each wearable device are input into the ensemble stacked LSTM-GRU deep learning architecture. This architecture comprises a sequential configuration of layers, including input, embedding, LSTM, hidden, GRU, flattening, and dense layers. Each layer passes learned representations to the next, enabling hierarchical feature extraction. The ensemble employs LSTM as the base learner and GRU as the meta learner, integrated using a stacking approach to leverage the strengths of both architectures. Each LSTM, hidden, and GRU layer contains 32 recurrent units, with the hyperbolic tangent (tanh) activation function applied to capture non-linear temporal patterns. In order to prevent overfitting, both recurrent dropout (rate of 0.1) and standard dropout (rate of 0.5) are incorporated across model layers, along with L2 regularization (coefficient of 0.001). The GRU output is flattened and passed to a fully connected dense layer that generates the final classification. For binary classification in discrete emotion recognition, a sigmoid activation function is used, while multi-class classification in dimensional emotion recognition is achieved using SoftMax activation. The output layer contains either two or three neurons, depending on the task type. The model is trained using the AdaMax optimizer and a sparse categorical cross-entropy loss function over 100 epochs with a batch size of 64. Early stopping, with a patience of five epochs, is employed based on validation loss to prevent overfitting and reduce computational overhead. As detailed in Table 1, the model structure and layer-wise output shapes are defined for the discrete emotion recognition system. Table 2 outlines a list of hyperparameters utilized in the study. The hyperparameter selection for the proposed ensemble LSTM-GRU architecture was guided by a combination of empirical experimentation and prior research on temporal modeling in physiological signal processing^1^. Robustness is ensured through multiple training iterations with varied random seeds and stratified data splits, where the model demonstrated consistent convergence and generalization performance.

**Table 2:** List of hypermeters utilized in the proposed study.

Hyperparameter	Values
No. of neurons	32
No. of hidden layers	1
Activation function	Tangent hyperbolic
Recurrent dropout	0.1
Dropout	0.5
L2 regularizer	0.001
Activation function	SoftMax (multi-class) & Sigmoid (binary-class)
Epoch	100
Batch size	64
monitor	val_loss
patience	5
restore_best_weights	False
optimizer	Adamax
loss	sparse_categorical_crossentropy
Number of neurons in dense layer	3 (multi-class) & 2 (binary-class)

**Fig. 3:**
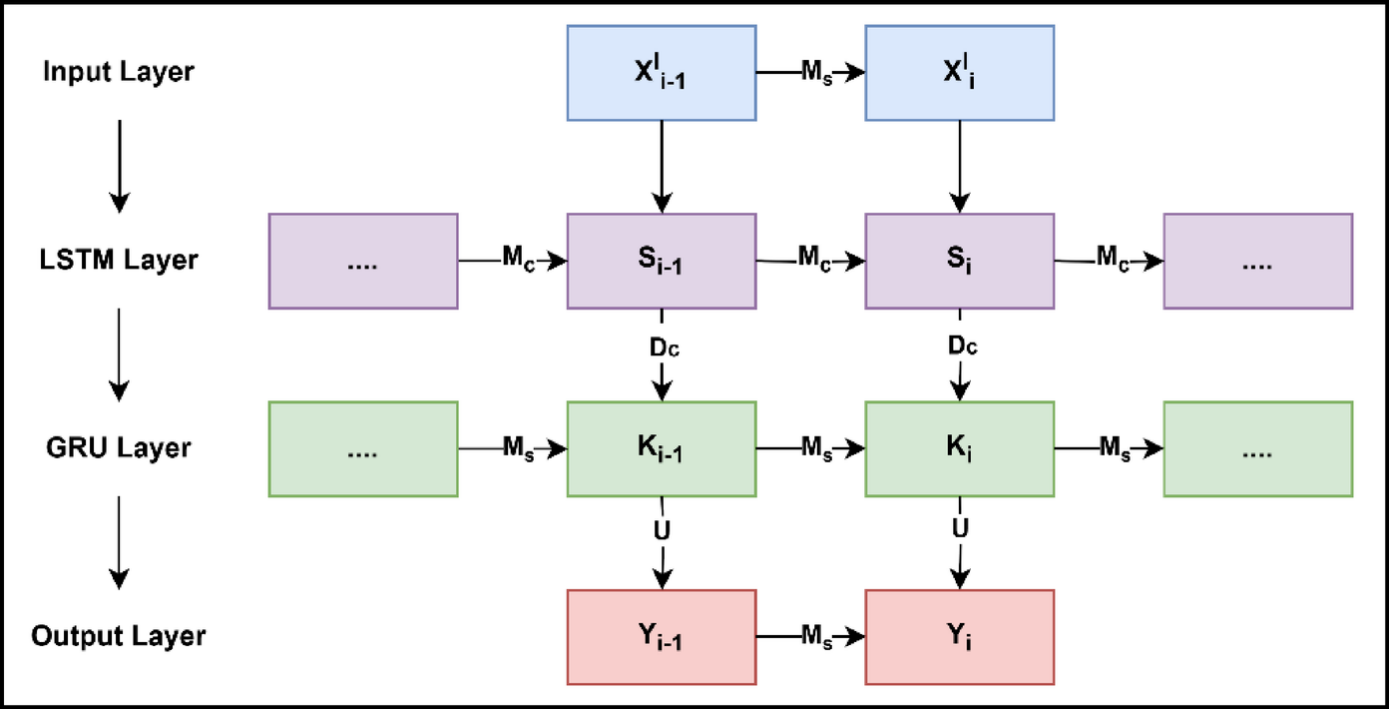
Illustrates the internal structure of the ensemble stacked LSTM-GRU model.

Figure 3 depicts the architecture of the ensemble stacked LSTM-GRU technique. Suppose $\:{\text{x}}_{\text{t}}^{\text{l}}$ denotes the input sequence of the signals, where $\:\text{l}\:$and $\:\text{t}\:$signify the emotion labels and time instance, respectively. The LSTM networks consist of a set of specialized gates designed to manage and retain temporal information effectively. The specialized gates are comprised of an input gate ($\:{\text{E}}_{\text{i}})$, a forget gate $\:{(\text{L}}_{\text{i}})$, and an output gate ($\:{\text{Y}}_{\text{i}}$). The information flow into, out of, and inside the memory cell is controlled by all three gates. Equation (3) represents the input gate, which controls the degree to which new information from the input is integrated into the memory cell at each time step. The forget gate determines which information from previous states should be retained or removed, represented by Eq. (4). The output gate decides the amount of memory information passed forward to the subsequent hidden state (Eq. 5). Lastly, Eq. (6) presents the candidate memory cell state that acts as a temporary memory component within the LSTM cell, capturing new input information at each iteration, which is then considered for integration into the LSTM’s overall memory.

3 $$\:\begin{array}{c}{\text{E}}_{\text{i}}=\sigma\:\left({\text{M}}_{\text{E}}{\text{x}}_{\text{i}}^{\text{l}}+{\text{D}}_{\text{E}}{\text{S}}_{\text{i}-1}+{\text{a}}_{\text{E}}\right)\end{array}$$

4 $$\:\begin{array}{c}{\text{L}}_{\text{i}}=\sigma\:\left({\text{M}}_{\text{L}}{\text{x}}_{\text{i}}^{\text{l}}+{\text{D}}_{\text{L}}{\text{S}}_{\text{i}-1}+{\text{a}}_{\text{L}}\right)\end{array}$$

5 $$\:\begin{array}{c}{\text{Y}}_{\text{i}}=\sigma\:\left({\text{M}}_{\text{Y}}{\text{x}}_{\text{i}}^{\text{l}}+{\text{D}}_{\text{Y}}{\text{S}}_{\text{t}-1}+{\text{a}}_{\text{Y}}\right)\end{array}$$

Here, the $\:{\text{M}}_{\text{E}}$, $\:{\text{M}}_{\text{L}}$, $\:{\text{M}}_{\text{Y}}$ are the weight matrix for the input, forget and output gate, respectively. The weight matrices are numerical parameters in the LSTM architecture which assign importance to input features, controlling information flow and capturing temporal patterns. The weight matrices are optimized during training to improve predictive accuracy. Similarly, the $\:{\text{D}}_{\text{E}},\:{\text{D}}_{\text{L}},\:\text{a}\text{n}\text{d}\:{\text{D}}_{\text{Y}}\:$are the bias vectors for the input, forget, and output gates, respectively. Bias vectors are added to the weighted sums of the inputs and the previous hidden state before being passed through the activation functions. These bias terms allow the gates to adjust their activation levels independently of the input values, enabling the network to shift decision boundaries and provide more flexibility in capturing complex patterns and temporal dynamics in the data. The $\:{\text{a}}_{\text{E}}$, $\:{\text{a}}_{\text{L}},\:{\text{a}}_{\text{Y}}$ are additional bias terms for the input, forget, and output gates, respectively. These biases further shift the activation outputs, allowing the gates to control the degree to which information from the input and the previous hidden state influences the current output. The inclusion of these bias terms ensures the model’s flexibility in learning non-linear relationships and better fits the training data. The $\:{\text{S}}_{\text{i}-1}$ is the hidden state at the preceding time step i-1, which stores the information from the past. $\:{\upsigma\:}$ is the sigmoid activation function, effectively controlling the flow of information between 0 and 1. The candidate cell state $\:{\text{C}}_{\text{i}}$ is given by the equation:

6 $$\:\begin{array}{c}{\text{C}}_{\text{i}}=tanh\left({\text{M}}_{\text{C}}{\text{t}}_{\text{i}}^{\text{l}}+{\text{D}}_{\text{C}}{\text{S}}_{\text{i}-1}+{\text{a}}_{\text{c}}\right)\end{array}$$

This candidate cell state $\:{\text{C}}_{\text{i}}$​ represents the potential new memory content at time step i that can be added to the cell state. $\:{\text{M}}_{\text{C}}$​ is the weight matrix for the candidate cell state. $\:{\text{D}}_{\text{C}}$ is the bias term for the candidate cell state, allowing for an additional shift in the activation function. $\:{\text{a}}_{\text{c}}$ is another bias term for the candidate cell state. LSTMs can effectively learn to preserve pertinent data across long sequences and lessen the issue of vanishing gradients, optimizing the three gates to allow for more accurate and robust predictions in sequential data analysis tasks. Since the proposed architecture is designed by combining the LSTM and GRU models, the output of the LSTM acts as an input for the GRU.

The architecture of GRU consists of a reset gate $\:{(\text{R}}_{\text{i}})$ and an update gate $\:{(\text{Z}}_{\text{i}})$, where the update gate functions as a simpler gate that is created by integrating the forget and input gates of the LSTM framework. The update gate updates the hidden gate’s status if the prior connection contains any significant information. Specifically, the reset gate determines the degree to which previous hidden state information is discarded, whereas the update gate controls how much information from the previous hidden state is carried forward. In the same way, the reset gate is constructed by combining the input and neglect gates of the LSTM model. This gate is responsible for preventing any information that is not significant in the model’s prediction from being passed on to the subsequent state. The mathematical expressions for reset gate and update gate are provided in the following equations.

7 $$\:\begin{array}{c}{\text{R}}_{\text{i}}=\sigma\:\left({\text{M}}_{\text{r}}{\text{x}}_{\text{i}}^{\text{l}}+\:{\text{U}}_{\text{r}}{\text{K}}_{\text{i}-1}+{\text{a}}_{\text{r}}\right)\end{array}$$

8 $$\:\begin{array}{c}{\text{Z}}_{\text{i}}=\sigma\:\left({\text{M}}_{\text{z}}{\text{x}}_{\text{i}}^{\text{l}}+\:{\text{U}}_{\text{z}}{\text{K}}_{\text{i}-1}+{\text{a}}_{\text{z}}\right)\end{array}$$

where, $\:{\text{M}}_{\text{r}}$, $\:{\text{M}}_{\text{z}}$ are the weight matrices to reset gate and update gate that control the contribution of the current input to the reset and update gates, $\:{\text{U}}_{\text{r}}$, $\:{\text{U}}_{\text{z}}$ provides the input to the hidden gate weight matrix and $\:{\text{a}}_{\text{r}}$, $\:{\text{a}}_{\text{z}}$ are the bias associated with both gates thereby adjusting activation thresholds, $\:{\upsigma\:}$ is the sigmoid function and $\:{\text{K}}_{\text{i}-1}$ is the hidden state at instant i-1. The states of both gates are employed to calculate hidden states at time i and $\:{\stackrel{\sim}{K}}_{i}$ is expressed in Eq. (9).

9 $$\:\begin{array}{c}{\stackrel{\sim}{\text{K}}}_{\text{i}}=\varphi\:\left({\text{M}}_{\text{s}}{\text{x}}_{\text{i}}^{\text{l}}+{\text{U}}_{\text{s}}\left({\text{R}}_{\text{i}}\odot\:{\text{K}}_{\text{i}-1}\right)+{\text{a}}_{\text{s}}\right)\end{array}$$

In this context, $\:{\upvarphi\:}$ represents the tanh function, while $\:{\text{M}}_{\text{s}}$ denotes the weight matrix, and $\:{\text{a}}_{\text{s}}\:$signifies the bias associated with the hidden state. The intermediate hidden state is computed by applying a hyperbolic tangent activation function to a combination of the current input, previous hidden state, and the reset gate. The reset gate modulates the previous hidden state, controlling how much past information is relevant. Weight matrices and scale the contributions of the current input and reset-modulated hidden state, respectively, while the bias vector adjusts activation thresholds. This intermediate state serves as a candidate for updating the current hidden state in the GRU layer. The following is a mathematical representation of the GRU layer’s output.

10 $$\:\begin{array}{c}{\text{K}}_{\text{i}}=\left(1-{\text{z}}_{\text{i}}\right)\odot\:\:{\text{K}}_{\text{i}-1}+{\text{z}}_{\text{i}}\:\odot\:\:{\stackrel{\sim}{\text{K}}}_{\text{i}}\end{array}$$

The current hidden state, $\:{\text{K}}_{\text{i}}\:$of the GRU is computed by combining the previous hidden state, $\:{\text{K}}_{\text{i}-1}$ and the intermediate candidate hidden state, $\:{\stackrel{\sim}{\text{K}}}_{\text{i}}$. The update gate $\:{\text{z}}_{\text{i}}$ regulates this combination, determining the amount of information retained from the past hidden state and the extent of new information from the intermediate candidate state. Specifically, $\:\left(1-{\text{z}}_{\text{i}}\right)$ scales the previous hidden state, emphasizing the retention of past information, while $\:{\text{z}}_{\text{i}}$ scales the candidate hidden state, incorporating new input information. This gating mechanism enables effective management of temporal dynamics within sequential data.

### Confidence interval

The confidence interval (CI) provides a statistical range within which the true value of a population parameter, such as a mean or proportion, is estimated to lie with a specified level of confidence. A widely used and suitable value for CI is the 95% confidence interval, suggesting that if the same sampling method is repeated numerous times, about 95% of the intervals calculated would contain the true population parameter^57^. The mathematical expression for calculating a confidence interval is:

11 $$\:CI=\stackrel{-}{x}\pm\:z\frac{s}{\sqrt{n}}$$

where $\:\stackrel{-}{x}\:$represents the sample mean, $\:z$​​ denotes the critical value from the standard normal distribution corresponding to the desired confidence level, s is the sample standard deviation, and n is the sample size. The CI provides insight into the precision and reliability of statistical estimates.

## Results and discussion

The experimentation of the ensemble stacked LSTM-GRU model employed in the design of a multi-modal emotion recognition system is performed on the Python platform using Keras, TensorFlow, matplotlib, NumPy, pandas, and similar other libraries. The performance and effectiveness of the ensemble stacked LSTM-GRU DL architecture for the emotion recognition system are assessed by the standard confusion matrix evaluation method. The confusion matrix parameters considered in the work are accuracy, F1 score, precision, and recall^58^. The pre-processed signal is split into training, testing, and validation datasets in the ratio of 60:20:20. The work employs the hold-out validation approach, which is widely favoured for its computational efficiency and ease of implementation when working with large datasets. The hold-out validation method entails dividing the dataset into separate training and testing subsets, with a substantial portion allocated for training and the remainder reserved for evaluation. This strategy enables efficient model assessment while avoiding the significant computational burden associated with iterative procedures such as cross-validation^59^. The designed emotion recognition system encompasses an experimental analysis for both the discrete and dimensional models of emotion detection. This is the first study conducted to assess the performance of three wearable devices for emotion recognition, utilizing both the discrete and dimensional models. The experimentation that has been conducted is anticipated to greatly broaden the possibilities for emotion recognition through the utilization of wearable devices. The results obtained are compared to prevailing methodologies in the literature and exhibit promising outcomes in wearable emotion recognition.

The work utilizes the publicly available EMOGNITION database to design and develop both the discrete and dimensional-based emotion recognition system. The database comprises 43 participants’ physiological recordings collected by three devices while watching various videos of emotional stimulations. All three devices, an EEG headband MUSE 2, a wristband Empatica E4, and a smartwatch Samsung Galaxy SM-R810, simultaneously record the participant’s physiological signals. All three devices enable real-time physiological signal tracking via advanced sensors and data processing, ensuring rapid and accurate acquisition, analysis, and visualization for healthcare professionals and researchers to gain insights into dynamic physiological states. The recorded physiological modalities are directly employed in DL algorithms to minimize the computational time and complexity associated with manually investigating signals through diverse preprocessing and feature extraction methods. The wearable Samsung Galaxy watch measures the subject’s ACC, BVP, GYRO, HR, PPI, and ROT signals, whereas the Empatica E4 records ACC, BVP, EDA, IBI, and SKT signals. The MUSE 2 device gathers the subjects’ ACC, EEG, and GYRO signals.

The database categorizes the collected signals into two analyzable groups: nine discrete emotions and a 2D VA dimensional model. Thus, the present work utilizes various modalities collected from three sensing devices to create and implement a system for recognizing emotions. The experimentation is performed using an ensemble-stacked LSTM-GRU DL architecture, fused to avail the benefits of both individual models. The subsequent section comprehensively discusses the results obtained for each of the designed emotion recognition systems.

**Fig. 4:**
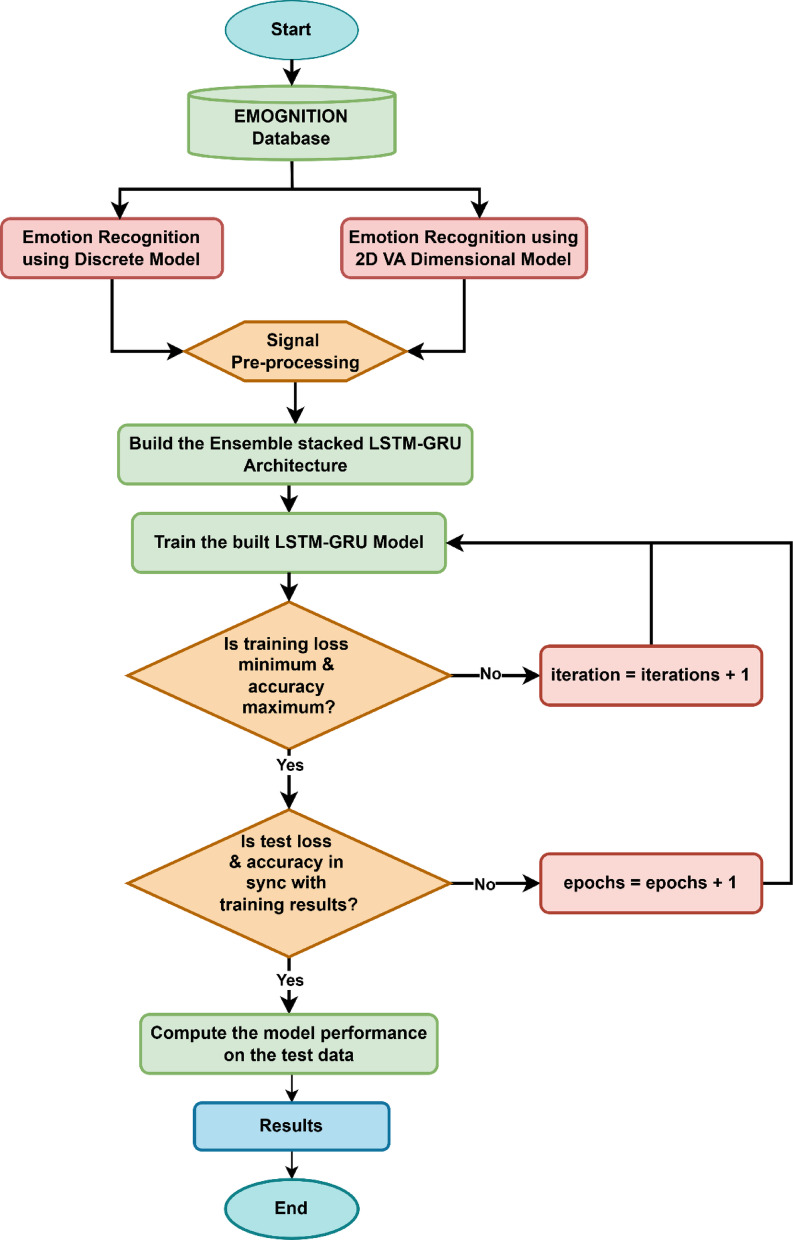
Illustrates the flow diagram for the proposed study.

Figure 4 illustrates a systematic workflow designed for emotion recognition using physiological signal data obtained from the EMOGNITION database. This approach employs two distinct emotion classification frameworks. The first framework utilizes a discrete model to categorize clearly defined emotional states, while the second framework applies a two-dimensional Valence-Arousal dimensional model to represent emotions across continuous dimensions. Initially, physiological signals undergo comprehensive preprocessing, including noise reduction, normalization, and feature extraction. Subsequently, an ensemble-stacked neural network architecture combining LSTM and Gated Recurrent Units (GRU) is developed and trained with the processed data. The training process proceeds iteratively until optimal results are achieved, which are characterized by minimal training loss and maximum accuracy. The model is then validated using test data to confirm consistency between training and testing performance metrics. Finally, upon successful validation, the model’s performance metrics are computed and documented as the results of the emotion recognition system.

### Performance analysis for the discrete emotion recognition system

Table 3 displays a comparative analysis of classification performance for the suggested emotion recognition system across the nine discrete emotions recorded using the three wearable devices. The results reveal that the MUSE 2 device and the Samsung Galaxy watch achieved an average classification accuracy of 99.41% and 99.14%, respectively. Similarly, the average precision, recall, and F1 score for the MUSE 2 device are 99.41%, 99.36%, and 99.38%, respectively. On the contrary, the Samsung Galaxy watch achieved 99.15%, 99.14%, and 99.14% average classification precision, recall, and F1 score for all nine discrete emotions. In contrast, the Empatica E4 device reported an average classification accuracy of 49.75%, showing a high rate of misclassification for the nine discrete emotions.

**Table 3:** The classification performance of the discrete model emotion recognition system obtained for three wearable devices.

Discrete Emotions	EEG Headband MUSE 2 Device				Empatica E4 wristband device				Samsung Galaxy Watch			
	Accuracy (%)	Precision (%)	Recall (%)	F1 Score (%)	Accuracy (%)	Precision (%)	Recall (%)	F1 Score (%)	Accuracy (%)	Precision (%)	Recall (%)	F1 Score (%)
Amusement	99.39	99.39	99.39	99.39	47.70	47.37	47.71	45.96	98.62	98.63	98.63	98.62
Angry	99.23	99.23	99.22	99.23	50.73	50.15	50.02	37.66	97.89	97.89	97.89	97.89
Sadness	99.94	99.94	99.94	99.93	50.03	25.03	50.00	33.34	97.93	97.94	97.93	97.93
Awe	99.61	99.61	99.61	99.61	49.85	24.85	50.00	33.26	99.22	99.22	99.22	99.22
Disgust	98.62	98.62	98.48	98.54	50.19	25.19	50.00	33.42	99.57	99.58	99.58	99.57
Enthusiasm	99.91	99.91	99.91	99.90	50.21	25.21	50.00	33.43	99.82	99.82	99.82	99.82
Fear	99.74	99.74	99.74	99.72	49.36	49.02	49.50	42.36	99.64	99.64	99.64	99.64
Liking	98.70	98.70	98.70	98.70	50.01	25.01	50.00	33.34	99.69	99.70	99.70	99.69
Surprise	99.53	99.53	99.23	99.41	49.65	24.65	50.00	33.18	99.93	99.93	99.93	99.93
Average	99.41	99.41	99.36	99.38	49.75	32.94	49.69	36.22	99.14	99.15	99.14	99.14

A comprehensive examination indicates that the Samsung Galaxy watch results demonstrate the discrete emotion “Surprise,” attaining the 99.93% maximum classification accuracy. The MUSE 2 headband attains 99.94% for the discrete emotion “Sadness,” whereas the Empatica E4 device records 50.73% as its highest classification accuracy for the discrete emotion “Angry.” Besides, the obtained results reveal the robustness of the signals collected using the Samsung Galaxy watch and the MUSE 2 by demonstrating consistent and accurate scores for all nine discrete emotions. The appropriateness of the ensemble stacked LSTM-GRU technique is observed in emotion recognition using the Samsung Galaxy watch and MUSE 2 wearable devices.

The wearable Samsung Galaxy Watch measures ACC, BVP, GYRO, HR, PPI, and ROT signals, while the Empatica E4 records ACC, BVP, EDA, IBI, and SKT, reflecting key differences in available physiological modalities. The BVP signal collected using the Samsung Galaxy Watch demonstrates higher reliability and contributes more effectively to classification performance than the BVP signal from the Empatica wristband. Additionally, the HR and PPI signals obtained from the Galaxy Watch show strong correlations with discrete emotional states, enhancing the discriminative power of the model. In contrast, the Empatica device lacks HR and PPI and relies on signal types that are more prone to noise and variability. Given that DL-based classification models require rich and diverse physiological inputs to achieve optimal performance, the lower accuracy associated with the Empatica device can be attributed to the absence of key cardiovascular signals. The HR and PPI signals captured by the Galaxy Watch thus play a crucial role in effective emotion recognition.

Additionally, the disparity in classification performance across Muse 2, Empatica E4, and Samsung Galaxy Watch can be attributed to several interrelated factors, primarily arising from a number of data samples, sensor modality, and signal characteristics. The Muse 2 and Galaxy Watch datasets comprise substantially more samples compared to the Empatica E4, enabling the models trained on them to generalize better and capture nuanced class-specific patterns with higher integrity. The Samsung Galaxy Watch and the Muse 2 headband, with their uniformly high data samples across modalities, required minimal to no resampling, thereby preserving the temporal fidelity of the signals and contributing positively to the overall model performance. In contrast, the Empatica E4 demonstrates reduced classification accuracy, primarily due to a limited sample size and reliance on BVP, IBI, EDA, and SKT signals. The susceptibility of such signal modalities to motion artifacts and environmental interference reduces signal reliability and compromises feature discriminability. Thus, necessitating the use of resampling of signals using the linear interpolation technique. The linear interpolation approach inherently assumes smooth transitions between consecutive data points, which may not accurately capture the dynamic nature of certain physiological signals, particularly those with low native sampling rates or abrupt transient changes, such as EDA and IBI signals. As a result, linear interpolation can introduce over-smoothing or artificial trends when aligning lower-frequency signals with higher-frequency signals. In addition, the IBI signal obtained from the Empatica wristband is susceptible to motion artifacts. It contains missing or irregular intervals due to signal loss or poor peak detection in the BVP signal. Nevertheless, it presents a pragmatic compromise between alignment precision and computational tractability, making it suitable for applications requiring synchronized multichannel input data.

The critical role of sensor modality and signal fidelity is further emphasized by the performance of Muse 2 and Galaxy Watch. The Muse 2 captures high-resolution EEG data through four electrodes (AF7, AF8, TP9, and TP10), allowing for detailed monitoring of frontal and temporal brain activity closely associated with emotional regulation. EEG frequency bands, particularly alpha, beta, and gamma, are well-established markers for valence and arousal states^60^. In addition to EEG, the device incorporates accelerometer and gyroscope sensors, enabling the extraction of motion-related features that support the differentiation of emotion-specific neural activity from physical movement artifacts and contribute to behavioural emotion context.

Similarly, the Samsung Galaxy Watch provides a diverse range of physiological signals relevant for emotion recognition. In particular, its BVP, HR, and PPI signals demonstrate strong correlations with discrete emotional states, offering valuable insight into cardiovascular dynamics linked to affective responses. The inclusion of inertial signals from ACC and GYRO sensors further enables fine-grained motion tracking, which can implicitly reflect emotional cues through posture and gesture changes. The combination of high-quality, temporally aligned biosignals across these modalities supports a robust framework for modeling emotional arousal. It enhances the accuracy and reliability of classification models in ecologically valid environments^61^. In comparison, although the Empatica E4 provides valuable physiological data, its performance is constrained by the limitations of its signal types. PPG-derived signals such as BVP and IBI are prone to noise and motion artifacts, often compromising the accuracy of derived metrics like HRV, which are critical for detecting arousal-related changes^62^. While EDA remains a recognized indicator of sympathetic nervous system activity, its phasic responses typically show a latency of 1–3 s after stimulus onset and are subject to high inter-individual variability in amplitude and timing^63^. These factors collectively result in a lower signal-to-noise ratio and reduced effectiveness in feature extraction, ultimately impacting the model’s ability to classify emotional states using E4 data accurately. Thus, the results reveal the significant impact of BVP, HR, PPI, ACC, GYRO, and ROT signals in emotion recognition and highlight the significance of the Samsung Galaxy Watch in emotion detection.

**Table 4:** Presents the confidence interval calculated for the accuracy parameter for three wearable devices.

Device	EEG Headband MUSE		Empatica E4 wristband device		Samsung Galaxy Watch	
Emotion	CI Lower (%)	CI Upper (%)	CI Lower (%)	CI Upper (%)	CI Lower (%)	CI Upper (%)
Amusement	98.60	98.64	46.88	48.52	99.34	99.44
Angry	97.86	97.92	50.43	51.03	99.21	99.25
Sadness	97.90	97.96	49.93	50.13	99.94	99.94
Awe	99.20	99.24	49.69	50.01	99.60	99.62
Disgust	99.56	99.58	50.07	50.31	98.60	98.64
Enthusiasm	99.81	99.83	50.01	50.41	99.91	99.91
Fear	99.63	99.65	48.61	50.11	99.74	99.74
Liking	99.68	99.70	49.91	50.11	98.68	98.72
Surprise	99.93	99.94	49.52	49.78	99.52	99.54

Table 4 demonstrates that the confidence interval values for both the EEG Headband MUSE and the Samsung Galaxy Watch exhibit high levels of accuracy and consistency, with confidence intervals ranging from approximately 97.86–99.94%. These narrow and high intervals indicate reliable and stable performance across multiple trials. In contrast, the Empatica E4 wristband displays significantly lower and more variable confidence intervals, ranging from 46.88 to 51.03%, reflecting reduced precision and greater uncertainty in its measurements. Overall, the results suggest that the EEG Headband MUSE and Samsung Galaxy Watch provide more dependable performance compared to the Empatica E4 device. Further these confidence interval values for different devices can be utilized for clinical significance of the model.

**Fig. 5:**
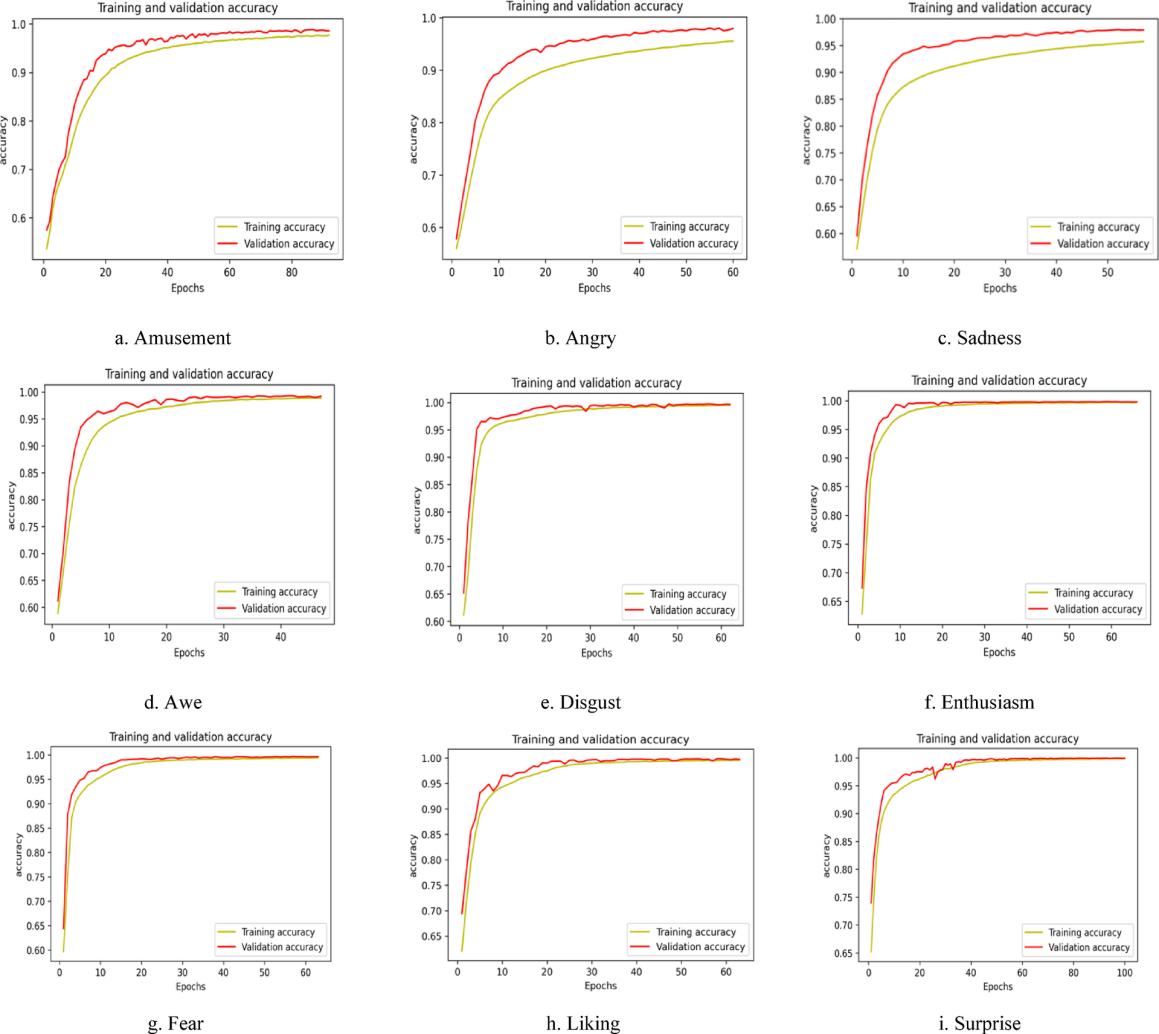
Graphical illustration of accuracy vs. epochs plot for nine discrete emotions recorded using Samsung Watch.

The work also incorporates a visual illustration of the obtained results for all three wearable devices. Figure 5 gives the accuracy vs. epochs plot for all nine discrete emotions (amusement, awe, enthusiasm, liking, surprise, anger, disgust, fear, and sadness) for the Samsung Galaxy watch. The designed ensemble stacked LSTM-GRU model is tested for 100 epochs. The close alignment between the training and validation accuracy indicates a well-fitted model, demonstrating its ability to generalize effectively on both the training and testing datasets. Thereby demonstrating the accuracy and precision of the classification score for all nine discrete emotions.

**Fig. 6:**
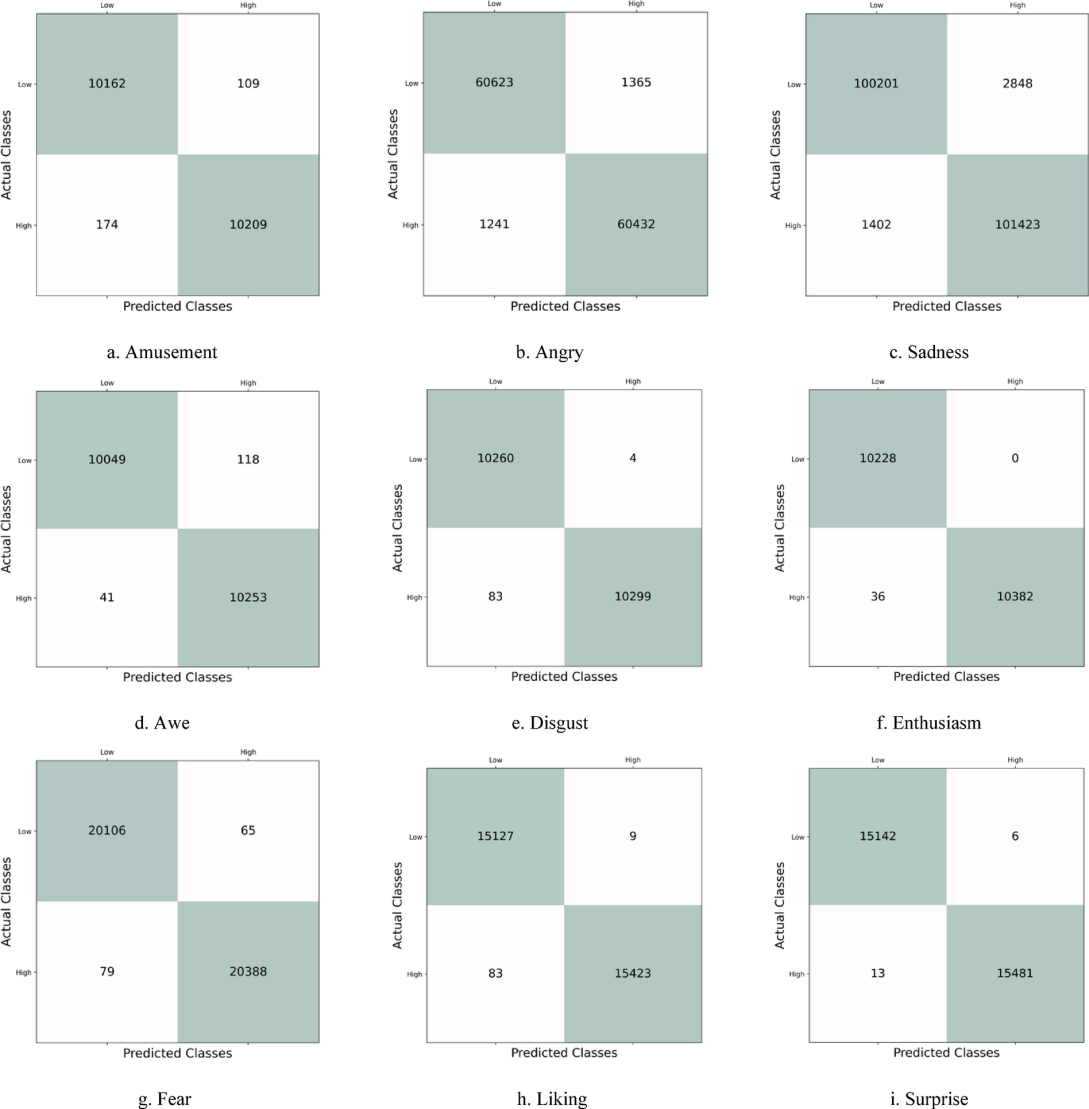
Graphical illustration of the confusion matrix for nine discrete emotions recorded using a Samsung Watch.

Figure 6 illustrates the confusion matrix associated with the binary classification of the discrete emotion recognition system implemented on the Samsung Galaxy watch. The diagonal entries represent the accuracy of classifications for both negative and positive instances. The notably low values in the lower-left and upper-right sections indicate the model’s effectiveness in accurately identifying false positive and negative data samples.

**Fig. 7:**
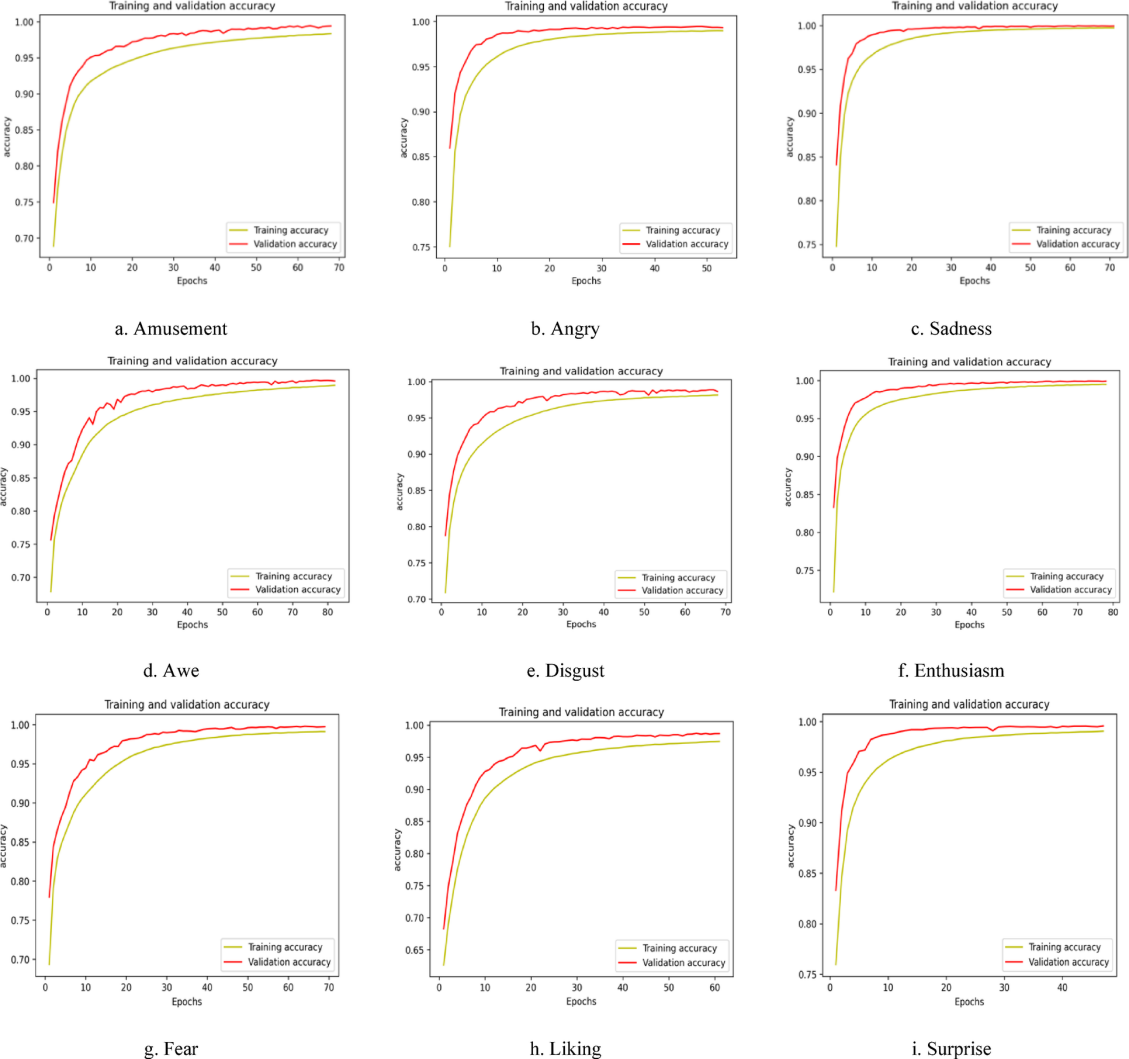
Graphical illustration of Accuracy vs. epochs plot for nine discrete emotions collected using EEG headband MUSE 2.

**Fig. 8:**
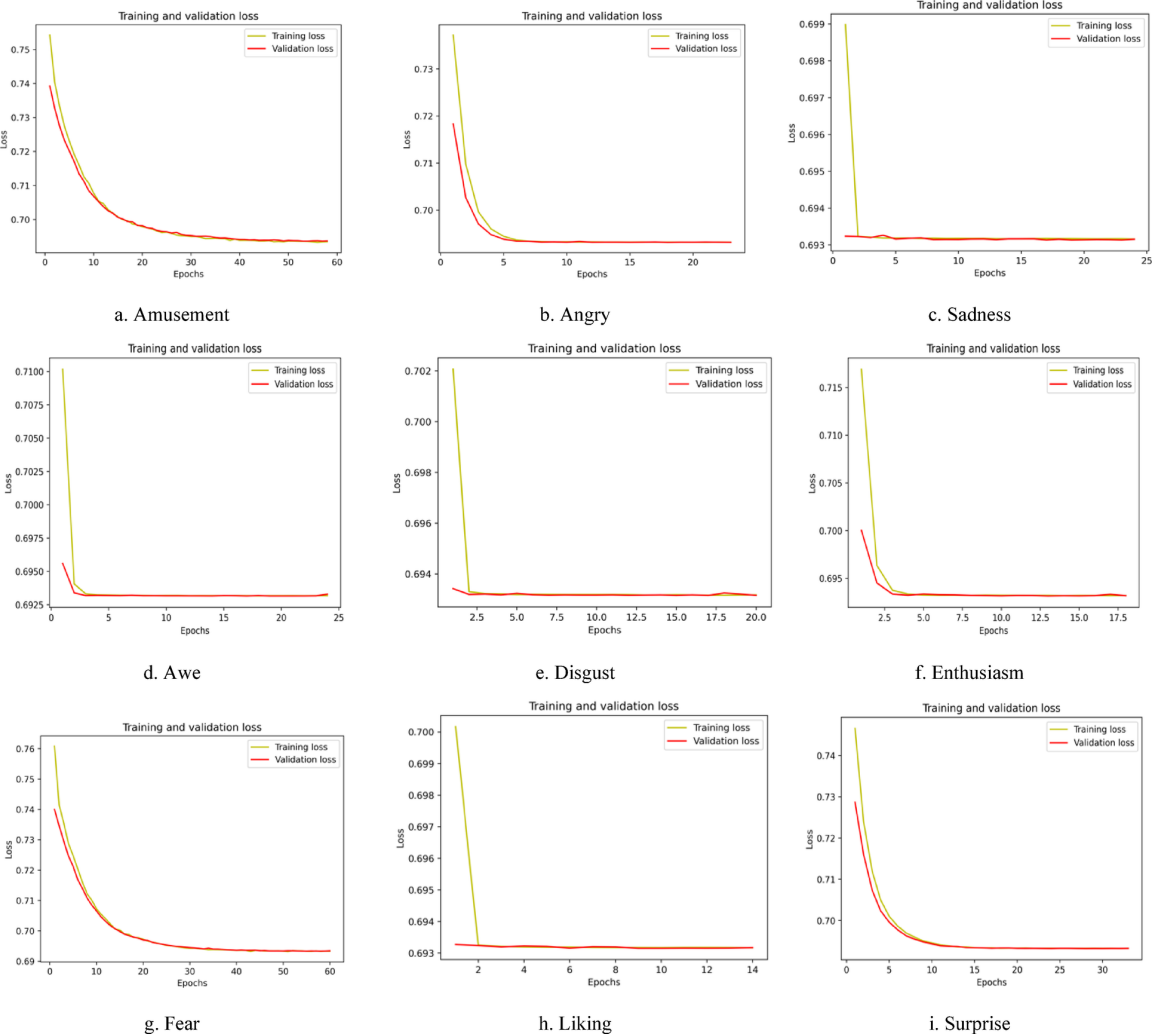
Graphical illustration of loss vs. epochs plot for nine discrete emotions recorded using the Empatica E4 wearable device.

Similarly, Fig. 7 shows the pictorial representation of the accuracy vs. epochs plot for the MUSE 2 headband with an epoch size of 100. The constrained computational capabilities of the Empatica E4 device necessitate representing the model’s performance through the loss vs. epochs plot, enabling a more insightful and meaningful evaluation of the designed model, shown in Fig. 8. The loss vs. epochs curve for the Empatica device demonstrates a high loss value, showing a significant scope for enhancements in the results.

Hence, the emotion recognition system designed using the Samsung Galaxy watch and the MUSE 2 headband demonstrates a promising outcome. Meanwhile, the outcomes obtained through the Empatica device necessitate enhancement.

### Emotion recognition for the 2D VA dimensional model: a performance analysis

The promising outcomes and increased wearability of the Samsung Galaxy Watch, in contrast to the MUSE 2 headband, motivate the experimentation of the designed LSTM-GRU model for the 2D VA dimensional framework. Table 5 compares the performance of the two affective dimensions of the Samsung Galaxy watch. The analysis reveals that the “Valence” affective dimension exhibits accuracy levels ranging from 94.17 to 99.94%. The amusement emotion records the lowest accuracy, while the highest accuracy is observed for the disgust emotion. In contrast, the “Arousal” affective dimension analysis reports the lowest classification accuracy of 61.26% for the “Surprise” emotion. The Amusement emotion achieves the uppermost classification accuracy, precision, recall, and F1 score of 94.95%, 94.96%, 93.86%, and 94.05%, respectively, for the “Arousal” affective dimension. Thus, the average classification accuracy for the 2D VA model is 97.81% and 72.94%, respectively.

**Table 5:** Performance analysis of a dimensional emotion recognition system on the Samsung galaxy watch.

Emotion	Valence Dimension				Arousal Dimension			
	Accuracy (%)	Precision (%)	Recall (%)	F1 Score (%)	Accuracy (%)	Precision (%)	Recall (%)	F1 Score (%)
Amusement	95.17	95.20	96.10	95.83	94.95	94.96	93.86	94.05
Angry	96.76	96.76	96.75	96.76	86.90	86.90	86.48	86.56
Sadness	95.11	95.13	95.13	94.98	72.17	72.27	71.77	72.04
Awe	99.78	99.78	99.82	99.71	69.80	69.93	70.17	70.05
Disgust	99.94	99.94	99.94	99.86	68.93	68.92	69.22	69.16
Enthusiasm	97.69	97.69	97.60	97.66	66.54	67.11	67.19	67.09
Fear	97.91	97.92	97.88	97.89	69.10	69.15	69.10	69.10
Liking	98.13	98.13	98.11	98.10	66.85	68.58	66.79	67.33
Surprise	99.88	99.88	99.84	99.87	61.26	61.52	61.80	62.05
Average	97.81	97.82	97.90	97.85	72.94	73.26	72.93	73.04

The difference in classification performance between the arousal and valence dimensions can be attributed to both the nature of the emotional stimuli and fundamental physiological and methodological factors. The film clips utilized in this study, designed to elicit nine discrete emotions such as awe, sadness, and liking, often evoke low to moderate arousal levels, which may not produce distinctly separable physiological patterns, thus reducing classification accuracy. In contrast, valence is typically represented more clearly by stimuli that evoke strongly positive or negative emotions, facilitating more reliable feature discrimination. Additionally, self-reports of valence tend to be more consistent across participants, whereas arousal ratings are generally more subjective and variable, contributing to potential label noise^64^. Furthermore, Feldman^65^ emphasized that physiological responses related to arousal exhibit considerable individual variability, leading to inconsistent physiological patterns. Valenza et al.^66^ also reinforced that accurate arousal recognition relies on complex nonlinear features often inadequately captured by peripheral signals alone. Similarly, another work highlighted that the relationship between valence and arousal is context-dependent and varies across individuals^67^. Consequently, the Samsung Galaxy Watch, which utilizes peripheral signals such as HR and PPI, provides high performance for valence detection while exhibiting reduced performance in arousal classification, reflecting these intrinsic physiological and methodological limitations.

**Table 6:** Presents the confidence interval of the accuracy parameter for the Samsung galaxy watch in a dimensional emotion recognition system.

Emotion	Valence Dimension		Arousal Dimension	
	CI Lower (%)	CI Upper (%)	CI Lower (%)	CI Upper (%)
Amusement	95.1290	95.2110	94.9081	94.9919
Angry	96.7261	96.7939	86.8354	86.9646
Sadness	95.0687	95.1513	72.0842	72.2558
Awe	99.7710	99.7890	69.7121	69.8879
Disgust	99.9353	99.9447	68.8414	69.0186
Enthusiasm	97.6499	97.7301	66.4464	66.6336
Fear	97.8702	97.9498	69.0215	69.1785
Liking	98.0905	98.1695	66.7575	66.9425
Surprise	99.8710	99.8890	61.1628	61.3572
Average	97.7702	97.8498	72.8553	73.0247

Table 6 shows that valence classification is consistently accurate, with scores ranging from 95.11 to 99.94% and narrow confidence intervals, indicating high reliability. In contrast, arousal accuracy is lower and more variable, ranging from 61.26 to 94.95%. Despite the large sample size ensuring narrow intervals, the average accuracy for arousal (72.94%) remains significantly lower than that of valence (97.82%), highlighting the need for improved arousal detection methods.

**Fig. 9:**
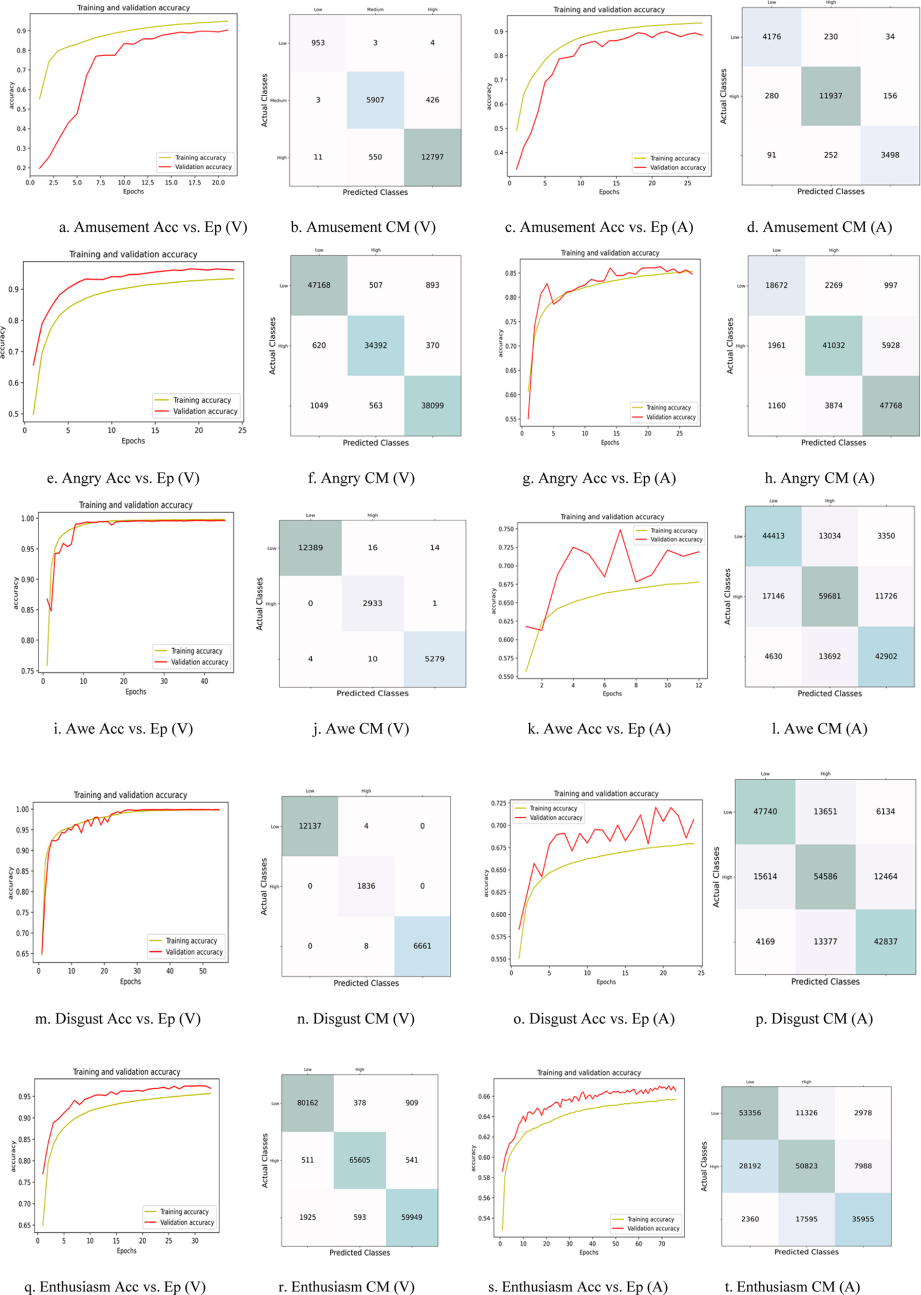

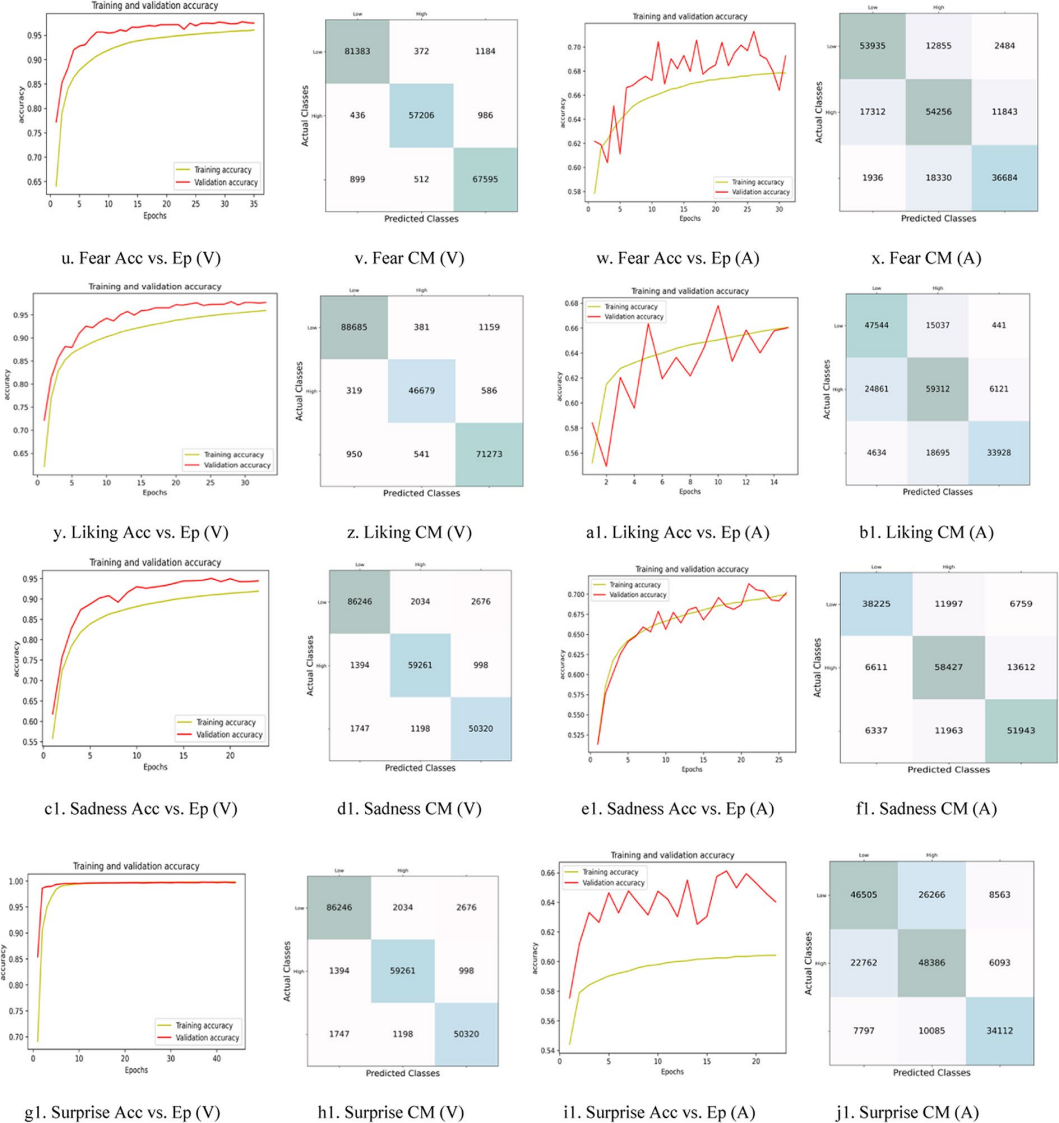
Graphical illustration of the Accuracy (Acc) vs. Epochs (Ep) plots and the Confusion Matrix (CM) plots for the 2D Valence (V)-Arousal (A) dimensional model for the nine emotions recorded using the Samsung Galaxy Watch.

Figure 9 graphically represents the accuracy vs. epochs plot and confusion matrix for the 2D VA dimensions for all nine emotions. The arousal dimension shows a poor fit for the “awe,” “fear,” “liking,” and “surprise” emotions, indicating the need for modification in the hyperparameters of the ensemble-stacked LSTM-GRU model.

Hence, the Samsung Galaxy watch is suitable for emotion detection in both the discrete and dimensional models. However, the outcomes for the arousal dimension of the 2D VA dimensional model need improvement. Modifying the developed LSTM-GRU model’s hyperparameters can enhance the outcomes of the arousal dimension.

### Comparative analysis of the proposed system with literature

Table 7 provides a comprehensive comparative analysis of the proposed emotion recognition system compared to the currently available emotion detection systems. It showcases critical performance metrics, highlighting the strengths and weaknesses of each approach. The state-of-the-art approaches do not utilize the identical physiological signals employed in the present study, thus rendering a direct comparison with the existing approaches somewhat intricate. However, the existing methods have potential for enhancement in emotion detection and identifying more discrete emotions. A work highlighting the use of EEG signals, examined using the MFCCs feature extraction method and classified using the SVM algorithm, reports an F1 score of 74.21%^24^. The constraint observed in a multi-modal analysis of GSR, HR, and PPG signals with a classification accuracy of 100% is the identification of only three discrete emotions^23^. Similarly, PPG signals are dominant in detecting the valence dimension, whereas the arousal dimension is influenced by ECG signals^68^. Another multi-modal signal analysis achieves 79% accuracy for the 2D VA model using STFT, VAE, and NB classifiers^11^. Several researchers have utilized the ECG signals for emotion recognition; a 7-layer CNN achieved 95.15% accuracy^27^, while a Poincare map and SVM approach reached 82.17% accuracy^28^. Another study employing EEG, ECG, and GSR with RF and KNN achieves 93.15% accuracy in valence-arousal and 91.30% in seven discrete emotions^35^, while^38^ achieves 91% accuracy using EEG and ECG with an eye-tracking gated strategy and ResNet-18. However, both studies encounter challenges in real-world wearability and emotional diversity. A 2-channel EEG and 3-channel GSR, PPG, and SKT-based system demonstrates 89.68% and 90.11% for Valence and arousal dimensions. Still, the binary classification limits the model’s ability to capture complex affective states, restricting its applicability in diverse emotional contexts^34^. A study using multi-channel EEG signals from the DEAP and SEED datasets^36^ achieved high accuracy in emotion recognition. Similarly, a 24-channel EEG-based study reported 99.81% accuracy using the TC-ResNet-18 CNN algorithm. Consequently, the heavy reliance on multi-channel EEG signals limits wearability, and the absence of additional physiological signals restricts additional performance improvements.

**Table 7:** Comparison of the proposed system’s performance metrics with existing methodologies.

Authors & year	Physiological Signals	Techniques	Results (%)	Discrete Emotions
Alqahtani et al.^24^, 2020	EEG, ECG, and EMG	PSD, Band-based Spectral EEG features, MFCCs (EEG), Spatial and spectral ECG features, statistical EMG feature, SVM	F1 score: 74.21 (MFCCs on EEG)	-
Domínguez et al.^23^, 2020	PPG, HR, GSR	time and frequency domain, and used RFE, SVM	Acc: 100 (GSR)	amusement, sadness, and neutral
Mert^25^, 2023	EEG, EOG, EMG, GSR, RESP, PPG, Temp	STFT, VAE, NB	Acc: 79 (V and A)	-
Ismail et al.^26^, 2022	ECG and PPG signals	AUBT (ECG), TEAP (PPG), SVM	Acc: 64.94 (V) (PPG) 68.75 (A) (ECG)	happy, sad, anger, fear, disgust, surprise, and neutral
Nita et al.^27^, 2022	ECG	Seven-layer CNN	95.15 (V), 85.56 (A) 77.54 (D)	-
Baghizadeh et al.^28^, 2020	ECG	Poincare map, SVM	82.17 (V) 78.07% (A)	-
Wei et al.^4^, 2018	ECG and EEG signals	SVM	Acc: 84.6	sadness, happiness, disgust, neutral, and fear
Baig et al.^2^, 2019	ECG signals	SVM classifier	Acc: 90	-
Ferinando et al.^30^, 2017	ECG	KNN	64.1 (V) 66.1(A)	-
Goshvarpour et al.^31^, 2017	ECG signals	SVM	80.24%	happy, sad, relaxed, and afraid,
Kanjo et al.^68^, 2019	EEG, GSR	CNN-LSTM model	Acc: 94.7 (V)	-
Wan et al.^34^, 2025	2-channel EEG and 3-channel PPG, GSR, SKT	Power spectral density (PSD), wavelet entropy, spectral asymmetry index (SASI), and differential entropy (DE), LightGBM	Acc: 89.68(V) 90.11 (A)	-
Kumar et al.^35^, 2025	EEG, ECG, and GSR	RF, KNN, LR	93.15 (VA) 91.30 (Basic emotions)	neutral, disgust, happy, surprised, anger, fear, and sad
Wang et al.^36^, 2024	EEG	Differential Entropy (DE), Bi-LSTM with attention mechanisms.	98.28 (SEED) 92.46 (DEAP)	-
Chen et al.^37^, 2024	EEG	TC-ResNet	99.81	-
Sedehi et al.^38^, 2024	EEG and ECG	Eye-tracking gated strategy, GC, ResNet-18 CNN	Acc: 91	-
Proposed Study	Samsung Watch: ACC, BVP, GYRO HR, PPI, ROT	Ensemble stacked LSTM-GRU model	Avg Acc: 99.14, 91.81 (V), 72.94 (A)	Nine Discrete emotions & 2D VA dimensions (Samsung Galaxy watch only)
	Empatica E4 wristband: ACC, BVP, EDA, IBI, Temp,		Avg Acc: 99.41	
	MUSE 2 headbands: EEG, GYRO, ACC		Avg Acc: 49.95	

Abbreviations: PPG: Photoplethysmography, Acc: Accuracy, HR: Heart rate, GSR: Galvanic Skin Response, RFE: Recursive Feature Elimination, EDA: Electro Dermal Activity, HRV: Heart Rate Variability, PSD: Power Spectral Density, MFCCs: Mel Frequency Cepstral Coefficients, SVM: Support Vector Machine, EOG: Electrooculogram, EMG: Electromyogram, RESP: Respiration belt, Temp: temp, V: Valence, A: Arousal, NB: Naïve Bayes, STFT: Short-time Fourier transform, VAE: Variational autoencoder, AUBT: Augsburg Biosignal Toolbox, TEAP: Toolbox for Extracting Emotional Features from Physiological Signals, BVP: Blood Volume Pressure, PPI: Peak-to-peak interval, GYRO: Gyroscope, ACC: Accelerometer, ROT: Rotation Signal, IBI: Interbeat interval, EEG: Electroencephalogram.

A similar trend of using ECG signals and traditional ML algorithms is evident in^1,2,30,31^, with^2^ demonstrating the highest performance and^4^ detecting the five discrete emotions. A hybrid CNN-LSTM DL technique designed using EEG and GSR signals reports a performance score of 94.7% for the valence dimension. Thus, the comparison reveals the need to detect significant discrete emotions and incorporate the emotional dimensional model into the analysis. Hence, the proposed work presents an experiment on various physiological signals recorded using the Samsung Galaxy Watch, MUSE 2 headband, and Empatica E4 wristband. The results obtained using the Samsung Galaxy watch and MUSE 2 headband demonstrate an average classification accuracy of 99.14% and 99.41%, respectively. Both devices could consistently recognize all nine discrete emotions and three affective dimensions, i.e., valence, arousal, and motivation. The high performance of the obtained results is attributed to the combined strengths of multiple signals and ensemble DL architecture. On the contrary, the Empatica wristband performs poorly, highlighting the importance of the combination of BVP, HR, and PPI signals recorded using the Samsung Galaxy watch. The poor performance can also be attributed to a lack of other essential Bio-signals such as HR, ECG, IBI, etc. Considering the promising findings from the Samsung Galaxy watch, the work investigates a comprehensive examination of the signals using the 2D affective dimensional model. The results show that the ensemble stacked LSTM-GRU technique is robust in the Valence dimension, yielding an average classification accuracy of 97.81%. However, the average classification accuracy for the arousal dimension is 72.94%, indicating scope for improvement. Hence, designing a multi-modal emotion recognition system using the ensemble stacked LSTM-GRU model demonstrates an effective and robust solution for emotion recognition.

## Conclusion

The proposed work aims to address the complex challenges associated with accurately recognizing emotions using wearable systems. The present work contributes to the enhanced capabilities of a wearable emotion detection system that utilizes an ensemble stacked deep learning architecture to investigate the signals for determining nine discrete emotions using binary classification. The three wearable devices used in the database are the EEG headband, MUSE 2, and Empatica E4. The results reveal an average classification accuracy of 99.14% and 99.41% using the Samsung Galaxy watch and MUSE 2 headband, respectively. The performance is also assessed using the 2D VA emotion detection model with the Samsung Galaxy device. The average classification accuracy for the 2D VA model is 97.81% and 72.94%, respectively. The developed system’s performance is critically compared to the state-of-the-art methods in the literature. The present study reveals that the proposed model holds great promise in enhancing the accuracy, robustness, and applicability of emotion recognition within wearable systems. A significant challenge in the proposed work lies in using a database gathered within a controlled laboratory setting. Unlike the real-world scenarios, the controlled laboratory environment has predefined repetitive assigned tasks, minimal noise, restricted movement, and precise sensor placement. Conversely, real-world scenarios introduce considerable variability and uncertainty, such as unpredictable user behavior, environmental noise, motion artifacts, and inconsistent sensor placement. Such factors can degrade signal quality and model reliability by introducing artifacts and noise into the recorded signals, thereby requiring the use of additional signal pre-processing techniques to ensure data integrity. In the future, an emotion-specific database can also be recorded using audio-video stimuli for a specific demography. Furthermore, a personalized and adaptive model could be constructed through the use of transfer learning or meta-learning techniques. The work could also integrate explainable AI approaches, which would also contribute to a better understanding of model decisions, thereby enhancing transparency and building user trust. In parallel, the inclusion of contextual information such as physical activity, environmental conditions, and location can significantly refine emotion recognition in real-world scenarios.

## Data Availability

The datasets used in this work are available at: https://dataverse.harvard.edu/dataset.xhtml?persistentId=doi:10.7910/DVN/R9WAF4.

## References

[1] Nandini, D. et al. Design of subject independent 3D VAD emotion detection system using EEG signals and machine learning algorithms. *Biomed. Signal Process. Control*. **85**, 104894 (2023).

[2] Baig, M. Z. & Kavakli, M. A survey on psycho-physiological analysis & measurement methods in multimodal systems. *Multimodal Technol. Interact.* **3** (2), 37 (2019).

[3] Ismail, S. N. M. S. et al. A systematic review of emotion recognition using cardio-based signals. *ICT Express*. **10** (1), 156–183 (2024).

[4] Wei, W. et al. Emotion recognition based on weighted fusion strategy of multichannel physiological signals. *Comput. Intell. Neurosci.* **2018** (1), 5296523 (2018). 30073024 10.1155/2018/5296523 PMC6057426

[5] Zeynali, M., Seyedarabi, H. & Afrouzian, R. Classification of EEG signals using transformer based deep learning and ensemble models. *Biomed. Signal Process. Control*. **86**, 105130 (2023).

[6] Ba, S. & Hu, X. Measuring emotions in education using wearable devices: A systematic review. *Comput. Educ.* **200**, 104797 (2023).

[7] Nandi, A. & Xhafa, F. A federated learning method for real-time emotion state classification from multi-modal streaming. *Methods* **204**, 340–347 (2022). 35314343 10.1016/j.ymeth.2022.03.005

[8] cheol Jeong, I., Bychkov, D. & Searson, P. C. Wearable devices for precision medicine and health state monitoring. *IEEE Trans. Biomed. Eng.* **66** (5), 1242–1258 (2018). 10.1109/TBME.2018.2871638 31021744

[9] Esmaeili, B. et al. The potential of wearable devices and mobile health applications in the evaluation and treatment of epilepsy. *Neurol. Clin.* **40** (4), 729–739 (2022). 36270687 10.1016/j.ncl.2022.03.005

[10] Xiao, W. et al. Deep interaction: wearable robot-assisted emotion communication for enhancing perception and expression ability of children with autism spectrum disorders. *Future Gener. Comput. Syst.* **108**, 709–716 (2020).

[11] Gualniera, L. et al. Emotional behavioural and autonomic dysregulation (EBAD) in Rett syndrome–EDA and HRV monitoring using wearable sensor technology. *J. Psychiatr. Res.* **138**, 186–193 (2021). 33862302 10.1016/j.jpsychires.2021.03.052

[12] Ehrhart, M. et al. A conditional Gan for generating time series data for stress detection in wearable physiological sensor data. *Sensors* **22** (16), 5969 (2022). 36015730 10.3390/s22165969 PMC9412645

[13] Iqbal, T. et al. A sensitivity analysis of biophysiological responses of stress for wearable sensors in connected health. *IEEE Access.* **9**, 93567–93579 (2021).

[14] Samson, C. & Koh, A. Stress monitoring and recent advancements in wearable biosensors. *Front. Bioeng. Biotechnol.* **8**, 1037 (2020). 32984293 10.3389/fbioe.2020.01037 PMC7492543

[15] Gedam, S. & Paul, S. A review on mental stress detection using wearable sensors and machine learning techniques. *IEEE Access.* **9**, 84045–84066 (2021).

[16] Can, Y. S., Arnrich, B. & Ersoy, C. Stress detection in daily life scenarios using smart phones and wearable sensors: A survey. *J. Biomed. Inform.* **92**, 103139 (2019). 30825538 10.1016/j.jbi.2019.103139

[17] Assabumrungrat, R. et al. Ubiquitous affective computing: A review. *IEEE Sens. J.* **22** (3), 1867–1881 (2021).

[18] Wang, F. et al. A geometric algebra-enhanced network for skin lesion detection with diagnostic prior. *J. Supercomput*. **81** (1), 327 (2024).

[19] Zhu, X. et al. Emotion recognition based on brain-like multimodal hierarchical perception. *Multimed. Tools Appl.* **83** (18), 56039–56057 (2024).

[20] Zhu, X. et al. A client–server based recognition system: Non-contact single/multiple emotional and behavioral state assessment methods. *Comput. Methods Programs Biomed.* **260**, 108564 (2025). 39732086 10.1016/j.cmpb.2024.108564

[21] Wang, R. et al. Multi-modal emotion recognition using tensor decomposition fusion and self-supervised multi-tasking. *Int. J. Multimed. Inform. Retr.* **13** (4), 39 (2024).

[22] Zhu, X. et al. A review of key technologies for emotion analysis using multimodal information. *Cogn. Comput.* **16** (4), 1504–1530 (2024).

[23] Domínguez-Jiménez, J. A. et al. A machine learning model for emotion recognition from physiological signals. *Biomed. Signal Process. Control*. **55**, 101646 (2020).

[24] Alqahtani, F., Katsigiannis, S. & Ramzan, N. Using wearable physiological sensors for affect-aware intelligent tutoring systems. *IEEE Sens. J.* **21** (3), 3366–3378 (2020).

[25] Mert, A. Modality encoded latent dataset for emotion recognition. *Biomed. Signal Process. Control*. **79**, 104140 (2023).

[26] Ismail, S. N. M. S., Aziz, N. A. A. & Ibrahim, S. Z. A comparison of emotion recognition system using electrocardiogram (ECG) and photoplethysmogram (PPG). *J. King Saud Univ.-Comput. Inform. Sci.* **34** (6), 3539–3558 (2022).

[27] Nita, S. et al. A new data augmentation convolutional neural network for human emotion recognition based on ECG signals. *Biomed. Signal Process. Control*. **75**, 103580 (2022).

[28] Baghizadeh, M. et al. A new emotion detection algorithm using extracted features of the different time-series generated from ST intervals poincaré map. *Biomed. Signal Process. Control*. **59**, 101902 (2020).

[29] Bulagang, A. F. et al. A review of recent approaches for emotion classification using electrocardiography and electrodermography signals. *Inf. Med. Unlocked*. **20**, 100363 (2020).

[30] Ferdinando, H., Seppänen, T. & Alasaarela, E. *Enhancing emotion recognition from ECG signals using supervised dimensionality reduction*. in *6th International Conference on Pattern Recognition Applications and Methods (ICPRAM)*. (SCITEPRESS Science And Technology Publications, 2017).

[31] Goshvarpour, A., Abbasi, A. & Goshvarpour, A. An accurate emotion recognition system using ECG and GSR signals and matching pursuit method. *Biomed. J.* **40** (6), 355–368 (2017). 29433839 10.1016/j.bj.2017.11.001 PMC6138614

[32] Muñoz-Saavedra, L. et al. Designing and evaluating a wearable device for affective state level classification using machine learning techniques. *Expert Syst. Appl.* **219**, 119577 (2023).

[33] Kacimi, Y. & Adda, M. From signals to emotion: affective state classification through Valence and arousal. *Procedia Comput. Sci.* **251**, 358–365 (2024).

[34] Wan, C. et al. Emotion recognition based on a limited number of multimodal physiological signals channels. *Measurement* **242**, 115940 (2025).

[35] Kumar, A. & Kumar, A. Human emotion recognition using machine learning techniques based on the physiological signal. *Biomed. Signal Process. Control*. **100**, 107039 (2025).

[36] Wang, J., Wang, Z. & Liu, G. Recording brain activity while listening to music using wearable EEG devices combined with bidirectional long Short-Term memory networks. *Alex. Eng. J.* **109**, 1–10 (2024).

[37] Chen, J. et al. Temporal convolutional network-enhanced real-time implicit emotion recognition with an innovative wearable fNIRS-EEG dual-modal system. *Electronics* **13** (7), 1310 (2024).

[38] Sedehi, J. F. et al. Multimodal insights into Granger causality connectivity: Integrating physiological signals and gated eye-tracking data for emotion recognition using convolutional neural network. *Heliyon*, **10**(16) (2024). 10.1016/j.heliyon.2024.e36411 PMC11381760 39253213

[39] Lee, J. & Yoo, S. K. Recognition of negative emotion using long short-term memory with bio-signal feature compression. *Sensors* **20** (2), 573 (2020). 31968700 10.3390/s20020573 PMC7014523

[40] Lee, M. S. et al. Fast emotion recognition based on single pulse PPG signal with convolutional neural network. *Appl. Sci.* **9** (16), 3355 (2019).

[41] Sepúlveda, A. et al. Emotion recognition from ECG signals using wavelet scattering and machine learning. *Appl. Sci.* **11** (11), 4945 (2021).

[42] Irshad, M. T. et al. Wearable-based human flow experience recognition enhanced by transfer learning methods using emotion data. *Comput. Biol. Med.* **166**, 107489 (2023). 37769461 10.1016/j.compbiomed.2023.107489

[43] Cosoli, G. et al. Measurement of multimodal physiological signals for stimulation detection by wearable devices. *Measurement* **184**, 109966 (2021).

[44] Zhao, B. et al. EmotionSense: Emotion recognition based on wearable wristband. in. *2018 IEEE SmartWorld, Ubiquitous Intelligence & Computing, Advanced & Trusted Computing, Scalable Computing & Communications, Cloud & Big Data Computing, Internet of People and Smart City Innovation (SmartWorld/SCALCOM/UIC/ATC/CBDCom/IOP/SCI)* (IEEE, 2018).

[45] Cui, S. et al. A stacking-based ensemble learning method for earthquake casualty prediction. *Appl. Soft Comput.* **101**, 107038 (2021).

[46] Umer, M. et al. Fake news stance detection using deep learning architecture (CNN-LSTM). *IEEE Access.* **8**, 156695–156706 (2020).

[47] Iyer, A. et al. CNN and LSTM based ensemble learning for human emotion recognition using EEG recordings. *Multimed. Tools Appl.* **82** (4), 4883–4896 (2023).

[48] Zeng, C. et al. Parking occupancy prediction method based on multi factors and stacked GRU-LSTM. *IEEE Access.* **10**, 47361–47370 (2022).

[49] Pudumalar, S. & Muthuramalingam, S. Hydra: An ensemble deep learning recognition model for plant diseases. *J. Eng. Res.* (2023).

[50] Islam, M. S. & Hossain, E. Foreign exchange currency rate prediction using a GRU-LSTM hybrid network. *Soft Comput. Lett.* **3**, 100009 (2021).

[51] Mohammed, A. & Kora, R. A comprehensive review on ensemble deep learning: Opportunities and challenges. *J. King Saud Univ.-Comput. Inform. Sci.* **35** (2), 757–774 (2023).

[52] Saganowski, S. et al. Emotion recognition using wearables: A systematic literature review-work-in-progress. in. *2020 IEEE International Conference on Pervasive Computing and Communications Workshops (PerCom Workshops)*. (IEEE, 2020).

[53] Saganowski, S. et al. Emognition dataset: Emotion recognition with self-reports, facial expressions, and physiology using wearables. *Sci. Data*. **9** (1), 158 (2022). 35393434 10.1038/s41597-022-01262-0 PMC8989970

[54] Kocaçınar, B. et al. NeuroBioSense: A multidimensional dataset for neuromarketing analysis. *Data Brief.* **53**, 110235 (2024). 38533115 10.1016/j.dib.2024.110235 PMC10964042

[55] Swana, E. F., Doorsamy, W. & Bokoro, P. Tomek link and SMOTE approaches for machine fault classification with an imbalanced dataset. *Sensors* **22** (9), 3246 (2022). 35590937 10.3390/s22093246 PMC9099503

[56] Cohen, W. W. & Carvalho, V. R. Stacked sequential learning. *CRF* **1**, 117 (2005).

[57] Hazra, A. Using the confidence interval confidently. *J. Thorac. Dis*. **9** (10), 4124–4129 (2017). 10.21037/jtd.2017.09.14 PMC5723800 29268424

[58] Nandini, D. et al. Improved patient-independent seizure detection using hybrid feature extraction approach with atomic function-based wavelets. *Iran. J. Sci. Technol. Trans. Electr. Eng.* **47** (4), 1667–1688 (2023).

[59] Eertink, J. J. et al. External validation: A simulation study to compare cross-validation versus holdout or external testing to assess the performance of clinical prediction models using PET data from DLBCL patients. *EJNMMI Res.* **12** (1), 58 (2022). 36089634 10.1186/s13550-022-00931-w PMC9464671

[60] Al-Nafjan, A. et al. Review and classification of emotion recognition based on EEG Brain-Computer interface system research: A systematic review. *Appl. Sci.* **7** (12), 1239 (2017).

[61] Gjoreski, M. et al. Continuous stress detection using a wrist device: in laboratory and real life. In *Proceedings of the 2016 ACM International Joint Conference on Pervasive and Ubiquitous Computing: Adjunct* 1185–1193. (Association for Computing Machinery: Heidelberg, Germany, 2016).

[62] Temko, A. Accurate heart rate monitoring during physical exercises using PPG. *IEEE Trans. Biomed. Eng.* **64** (9), 2016–2024 (2017). 28278454 10.1109/TBME.2017.2676243

[63] Boucsein, W. *Electrodermal Activity* (Springer Science & Business Media, 2012).

[64] Alarcão, S. M. & Fonseca, M. J. Identifying emotions in images from Valence and arousal ratings. *Multimed. Tools Appl.* **77** (13), 17413–17435 (2018).

[65] Feldman, L. A. Valence focus and arousal focus: individual differences in the structure of affective experience. *J. Personal. Soc. Psychol.* **69** (1), 153 (1995).

[66] Valenza, G., Lanata, A. & Scilingo, E. P. The role of nonlinear dynamics in affective Valence and arousal recognition. *IEEE Trans. Affect. Comput.* **3** (2), 237–249 (2012).

[67] Kuppens, P. et al. The relation between Valence and arousal in subjective experience. *Psychol. Bull.* **139** (4), 917–940 (2013). 23231533 10.1037/a0030811

[68] Kanjo, E., Younis, E. M. & Ang, C. S. Deep learning analysis of mobile physiological, environmental and location sensor data for emotion detection. *Inform. Fusion*. **49**, 46–56 (2019).

